# Tomato UDP-Glucose Sterol Glycosyltransferases: A Family of Developmental and Stress Regulated Genes that Encode Cytosolic and Membrane-Associated Forms of the Enzyme

**DOI:** 10.3389/fpls.2017.00984

**Published:** 2017-06-09

**Authors:** Karla Ramirez-Estrada, Nídia Castillo, Juan A. Lara, Monserrat Arró, Albert Boronat, Albert Ferrer, Teresa Altabella

**Affiliations:** ^1^Plant Metabolism and Metabolic Engineering Program, Centre for Research in Agricultural Genomics (CRAG) (CSIC-IRTA-UAB-UB)Barcelona, Spain; ^2^Department of Biochemistry and Physiology, Faculty of Pharmacy and Food Sciences, University of BarcelonaBarcelona, Spain; ^3^Department of Biochemistry and Molecular Biomedicine, Faculty of Biology, University of BarcelonaBarcelona, Spain; ^4^Department of Biology, Healthcare and the Environment, Faculty of Pharmacy and Food Sciences, University of BarcelonaBarcelona, Spain

**Keywords:** Arabidopsis, conjugated sterols, fluorescence recovery after photobleaching, sterol glycosylation, stress response, *Solanum lycopersicum*, subcellular localization

## Abstract

Sterol glycosyltransferases (SGTs) catalyze the glycosylation of the free hydroxyl group at C-3 position of sterols to produce sterol glycosides. Glycosylated sterols and free sterols are primarily located in cell membranes where in combination with other membrane-bound lipids play a key role in modulating their properties and functioning. In contrast to most plant species, those of the genus *Solanum* contain very high levels of glycosylated sterols, which in the case of tomato may account for more than 85% of the total sterol content. In this study, we report the identification and functional characterization of the four members of the tomato (*Solanum lycopersicum* cv. Micro-Tom) *SGT* gene family. Expression of recombinant SlSGT proteins in *E. coli* cells and *N. benthamiana* leaves demonstrated the ability of the four enzymes to glycosylate different sterol species including cholesterol, brassicasterol, campesterol, stigmasterol, and β-sitosterol, which is consistent with the occurrence in their primary structure of the putative steroid-binding domain found in steroid UDP-glucuronosyltransferases and the UDP-sugar binding domain characteristic for a superfamily of nucleoside diphosphosugar glycosyltransferases. Subcellular localization studies based on fluorescence recovery after photobleaching and cell fractionation analyses revealed that the four tomato SGTs, like the Arabidopsis SGTs UGT80A2 and UGT80B1, localize into the cytosol and the PM, although there are clear differences in their relative distribution between these two cell fractions. The *SlSGT* genes have specialized but still largely overlapping expression patterns in different organs of tomato plants and throughout the different stages of fruit development and ripening. Moreover, they are differentially regulated in response to biotic and abiotic stress conditions. *SlSGT4* expression increases markedly in response to osmotic, salt, and cold stress, as well as upon treatment with abscisic acid and methyl jasmonate. Stress-induced *SlSGT2* expression largely parallels that of *SlSGT4*. On the contrary, *SlSGT1* and *SlSGT3* expression remains almost unaltered under the tested stress conditions. Overall, this study contributes to broaden the current knowledge on plant SGTs and provides support to the view that tomato SGTs play overlapping but not completely redundant biological functions involved in mediating developmental and stress responses.

## Introduction

**FIGURE 1:**
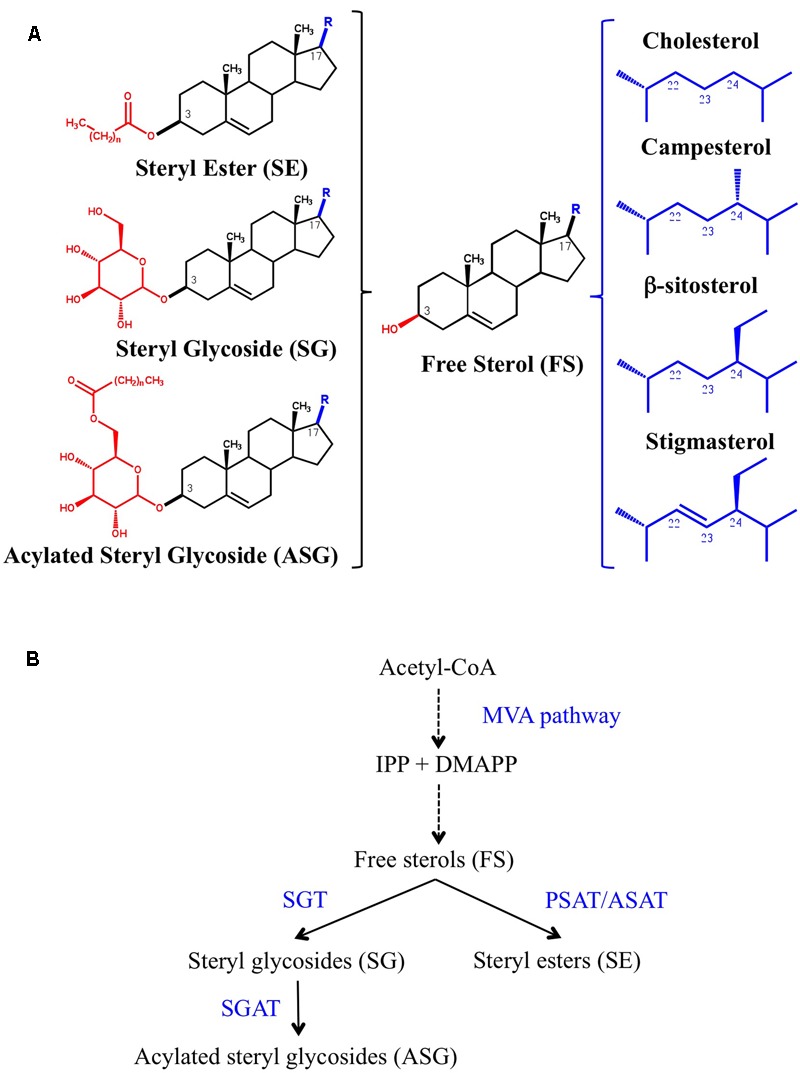
**(A)** The chemical structure of free (FS) and conjugated sterols (SE, SG, and ASG) is shown on the left side. The aliphatic side chain (R) attached to the C-17 position of cholesterol, the major sterol in animals, and campesterol, β-sitosterol and stigmasterol, the most abundant plant sterols, is shown on the right side. **(B)** Biosynthesis of conjugated sterols. FS derive from isopentenyl diphosphate (IPP) and dimethylallyl diphosphate (DMAPP) produced by the mevalonic acid (MVA) pathway. Dashed arrows indicate multiple steps. The position of the enzymes phospholipid:sterol acyltransferase (PSAT), acyl-CoA:sterol acyltransferase (ASAT), UDP-glucose:sterol glycosyltransferase (SGT), and steryl glycoside acyltransferase (SGAT) is indicated.

Plants contain a complex mixture of more than 100 different sterols consisting of three major species, namely β-sitosterol (the most prevalent one), stigmasterol and campesterol, and a variety of minor sterols that are biosynthetic precursors of main sterols. Cholesterol, whose biosynthetic pathway in plants has only very recently been elucidated (Sonawane et al., 2016), is also a major sterol in some members of the *Solanaceae* family (Moreau et al., 2002; Schaller, 2003; Benveniste, 2004). Sterols occur in free form (FS), with a free β-hydroxyl group at C-3 position on the sterol backbone, and conjugated as sterol esters (SEs), sterol glycosides (SGs) and acyl sterol glycosides (ASGs) (**Figure 1A**). SE contain a fatty acid group attached through an ester linkage to the hydroxyl group at C-3, whereas in SG the hydroxyl group is linked through a glycosidic bond to a sugar moiety (usually a single glucose residue), which increases the hydrophilicity of the sterol moiety. In turn ASG are derivatives of SG in which the hydroxyl group at C-6 position of the sugar moiety is esterified with a fatty acid (Moreau et al., 2002; Benveniste, 2004). All these sterol forms are enzymatically interconvertible, with FS occupying a branch point position in the metabolism of conjugated sterols (**Figure 1B**). Steryl esters are stored in cytoplasmic lipid bodies and are suggested to serve as a reservoir to maintain the levels of FS in cell membranes within the physiological range (Bouvier-Navé et al., 2010). On the contrary, FS, SG, and ASG are primarily located in the plasma membrane (PM), where in combination with other lipids play an essential role in maintaining proper membrane structure and functioning (Schaller, 2004). Interestingly, FS, SG, and ASG are unevenly distributed in the PM, being particularly enriched in the detergent-resistant membrane (DRM) fraction, so-called for the experimental condition used for its isolation method (Laloi et al., 2007; Lefebvre et al., 2007; Furt et al., 2010). So far it is unclear whether or not DRM reflects some pre-existing structure or organization reminiscent of the lipid rafts found in the PM of animal cells (Tanner et al., 2011; Malinsky et al., 2013). The presence of both free and conjugated sterols has been reported also in the phloem sap, where cholesterol is the dominating sterol and about half of the sterol pool is glycosylated (Behmer et al., 2013).

Changes in the relative proportions of sterols alter membrane fluidity and permeability (Roche et al., 2008; Grosjean et al., 2015) and hence regulate different membrane functions such as simple and carrier-mediated diffusion, active transport across the membrane, and the activity of membrane-associated proteins (Carruthers and Melchior, 1986; Cooke and Burden, 1990; Grandmougin-Ferjani et al., 1997). The importance of sterols in determining membrane biophysical properties also gives PM sterol levels a prominent role in the adaptive responses of plants to different types of abiotic and biotic stress, including tolerance to thermal stress (Hugly et al., 1990; Beck et al., 2007; Senthil-Kumar et al., 2013), drought (Posé et al., 2009; Kumar et al., 2015), metal ions (Urbany et al., 2013; Wagatsuma et al., 2015), H_2_O_2_ (Wang H. et al., 2012), and bacterial or fungal pathogens (Griebel and Zeier, 2010; Wang K. et al., 2012; Kopischke et al., 2013). In addition to their key structural function, sterols also play essential roles in modulating plant growth and development, not only because campesterol is the biosynthetic precursor of the brassinosteroid hormones (Yokota, 1997) but also because changes in sterol composition directly affect a number of cell processes, such as vascular and stomatal patterning (Jang et al., 2000; Carland et al., 2002; Qian et al., 2013), cell division, expansion and polarity (He et al., 2003; Men et al., 2008), cell-to-cell connectivity (Grison et al., 2015), hormonal regulation (Souter et al., 2002; Kim et al., 2010), vacuole trafficking (Li et al., 2015), cell wall formation (Schrick et al., 2012), pollen viability (Ischebeck, 2016) and even proper plastid development (Babiychuk et al., 2008; Kim et al., 2010; Itkin et al., 2012; Manzano et al., 2016). The specific contribution of glycosylated sterols, particularly of SG, to these processes is far from being fully understood, although there is increasing evidence supporting an important role of the ratio of conjugated to free sterol forms in regulating the properties of the cell membranes (Moreau et al., 2002; Grille et al., 2010; Grosjean et al., 2015) and therefore of different PM-associated processes like plant adaptation to biotic and abiotic stress conditions (Lynch and Steponkus, 1987; Palta et al., 1993; Uemura and Steponkus, 1994; Moreau et al., 2002; Minami et al., 2009; Mishra et al., 2013; Li et al., 2014; Pandey et al., 2014; Tarazona et al., 2015; Saema et al., 2016; Singh et al., 2016; Takahashi et al., 2016), signaling and transport, and recruitment of proteins to specific membrane subcompartments (Zauber et al., 2014). SGs have also been suggested to serve as primers for ceramide glycosylation (Lynch et al., 1997) and cellulose biosynthesis (Peng et al., 2002; Li et al., 2014), but whether SGs are primers for cellulose synthesis *in vivo* still remains an open question (Schrick et al., 2012).

Glycosylated sterols are widespread among plants, but the total content and the relative proportions of these compounds may vary greatly and are heavily dependent on the plant species, organs and growth conditions. Moreover, the sterol profile of glycosylated sterols does not always reflect the total sterol composition in the same plant tissue, which indicates preferential glycosylation of specific sterol species (Moreau et al., 2002; Nyström et al., 2012). In most plant species, SGs and ASGs are minor components of the total sterol fraction (Wojciechowski, 1991; Nyström et al., 2012). However, plants of the genus *Solanum* are a remarkable exception due to their extremely high content of glycosylated sterols (Duperon et al., 1984; Furt et al., 2011; Nyström et al., 2012), which in the case of tomato leaves and fruits accounts for more than 85% of total sterols (Duperon et al., 1984; Whitaker, 1988; Whitaker and Gapper, 2008). Moreover, the content and the profile of free and conjugated sterols, including the glycosylated forms, change dramatically during tomato fruit ripening and also during fruit chilling and after re-warming (Whitaker, 1988, 1991, 1994). So far the biological significance of these changes and the extremely high content of glycosylated sterols in certain *Solanaceae* is not completely understood, although it has been suggested that it might be necessary to protect cell membrane integrity against the disruptive effect of steroidal glycoalkaloids present in these plant species (Steel and Drysdale, 1988; Keukens et al., 1995; Blankemeyer et al., 1997). In fact, the levels of these compounds are under tight transcriptional control (Cárdenas et al., 2016). In the case of tomato, it may explain why tomato tissue is able to withstand the high concentration of α-tomatine (Steel and Drysdale, 1988), a bioactive steroidal glycoalkaloid involved in plant defense against a broad range of phytopathogens that accumulates in tomato tissues (Friedman, 2002; Iijima et al., 2013).

Sterol glycosides are synthesized by UDP-glucose:sterol glucosyltransferases (SGT; EC 2.4.1.173), which catalyze the transfer of a glucose residue from UDP-glucose to the free hydroxyl group at position C-3 of FS. The presence of SGT activity has been detected in a number of higher plants and the reaction has been suggested to be associated primarily with cell membranes (Grille et al., 2010; Chaturvedi et al., 2011; Li et al., 2014; Tiwari et al., 2014; Zauber et al., 2014) although the occurrence of soluble cytosolic SGTs has also been described (Madina et al., 2007; Grille et al., 2010; Li et al., 2014). On the contrary, SGT isozymes have only been cloned and functionally characterized in a handful of plant species, including *Arabidopsis thaliana* (Warnecke et al., 1997; DeBolt et al., 2009; Stucky et al., 2015), *Withania somnifera* (Sharma et al., 2007; Chaturvedi et al., 2012), *Avena sativa* (oat) (Warnecke et al., 1997), *Gossypium hirsutum*, Huamian99 (cotton) (Li et al., 2014) and *Gymnema sylvestre* R.Br. (Tiwari et al., 2014), of which only *W. somnifera* belongs to the *Solanaceae* family. These studies have shown that plants contain small gene families encoding SGT isozymes that are likely to play specialized functions. Thus, the genes coding for the two Arabidopsis SGTs, referred to as UGT80A2 and UGT80B1, are differentially expressed (DeBolt et al., 2009) and the encoded enzymes display distinct substrate preferences toward the major plant sterols as demonstrated by the analysis of the sterol composition of SG and ASG fractions in seeds of *ugt80A2* and *ugt80B1* knock-out mutants and the biochemical characterization of the recombinant UGT80A2 and UGT80B1 enzymes (Stucky et al., 2015). Moreover, the four members of the *W. somnifera SGT* gene family are also differentially induced upon heat and cold stress and treatment with jasmonic acid (JA) and salicylic acid (SA) (Chaturvedi et al., 2012), and in line with the latter observation, a distinct response pattern to heat shock has been reported for the two cotton *GhSGT* genes, which code for isozymes with different biochemical properties and a likely differential subcellular localization (Li et al., 2014).

On the basis of all the above considerations and as a first step toward the elucidation of the biological function of SGTs in tomato growth and development as well as in the adaptation to stress conditions, we undertook this study aimed at characterizing the *SGT* gene family in the dwarf tomato variety *Solanum lycopersicum* cv. Micro-Tom.

## Materials and Methods

### Plant Material, Growth Conditions, and Treatments

*Solanum lycopersicum* cv. Micro-Tom adult plants were grown on pots filled with a mixture of peat (Klasmann TS2), vermiculite and perlite (2:1:1) in a greenhouse set at 25°C. Seedlings were grown axenically into a growth chamber set for long day conditions (16 h light/8 h darkness) at an irradiance of 150 μmol^-2^s^-1^ and 24°C. In the latter case, seeds were rinsed by immersion in sterile water for 30 min and surface-sterilized by treatment with an antifungal solution of 0.3% (w/v) Captan50 (Bayer) for 5 min. Seeds were rinsed again with sterile water, disinfected for 30 min with a solution consisting of 40% (v/v) sodium hypochlorite and 0.1% (v/v) Tween 20, and washed again with sterile water. The sterilized seeds were then sown in glass jars containing solid (0.8% agar) 0.5x Murashige and Skoog (MS) basal salts medium (pH 5.8) supplemented with Gamborg B5 vitamins and sucrose 3% (w/v). Jars were kept in darkness at 24°C for 2 days and transferred to the growth chamber. After 12 days, pools of five seedlings were transferred to glass jars containing 30 mL of previously described MS liquid medium lacking sucrose and allowed to grow under the same conditions for 1 week more. Then, the growth medium was replaced by new MS liquid medium supplemented with the desired effectors: 200 mM mannitol, 150 mM NaCl, 0.1 mM abscisic acid (ABA), 0.5 mM SA, 1 μM flagellin 22 or 0.5 mM methyl jasmonate (MeJA). For wounding experiments leaves of seedlings in the MS liquid medium were injured with forceps, whereas for cold treatment seedlings were transferred to a growth chamber set at 4°C.

### Cloning of SlSGT cDNA Sequences

The entire ORFs encoding SlSGT1, SlSGT2, SlSGT3, and SlSGT4 were amplified by PCR using high fidelity AccuPrime Taq DNA polymerase (Invitrogen), specific primer pairs encompassing the corresponding start and stop codons (Supplementary Table S1) and cDNA prepared from RNA obtained from red fruit pericarp tissue samples (SlSGT1, SlSGT2, and SlSGT3) or seedlings treated with ABA (SlSGT4) as described above. Total RNA was extracted from tissue samples (100 mg fresh weight) using the PureLink RNA Mini Kit (Life Technologies). The RNA samples were treated with DNase I (DNA-free kit; Life Technologies) in a final reaction volume of 25 μL, and cDNA was synthesized from 1 μg of total RNA using SuperScript III Reverse Transcriptase (Invitrogen) and oligo(dT) primers according to the manufacturer’s instructions. The CACC sequence was added to the 5′ end of the forward primers to facilitate directional Gateway recombination-based cloning of the amplified sequences into pENTR/D-TOPO vector using the TOPO cloning kit (Invitrogen). The cDNAs in the resulting pENTR-SlSGT plasmids were sequenced to exclude the presence of amplification mutations.

### Heterologous Expression of GST-SlSGT Fusion Proteins in *E. coli*

The SlSGT ORFs were amplified by PCR using the primer pairs indicated in Supplementary Table S1, pENTR-SlSGT plasmids as templates and AccuPrime Taq DNA polymerase. A *Bam*HI restriction site was included in the sequence of reverse primers used for SlSGT1, SlSGT2, and SlSGT3 ORF amplification whereas a *Sma*I restriction site was included in the reverse primer for amplification of the SlSGT3 ORF. The resulting products were digested with either *Bam*HI or *Sma*I and cloned into the corresponding sites of the pGEX-3X-NotI vector previously digested with *Not*I and blunt-ended by treatment with nuclease S1, as described in Arró et al. (2014). All constructs were sequenced to confirm the in-frame sequence fusions. The resulting recombinant plasmids and the pGEX-3X-NotI empty vector were transformed into competent *E. coli* BL21 (DE3) cells harboring plasmid pUBS520, which carries the gene coding for the tRNA of rare arginine codons AGG and AGA. The correct fusion between the GST and SlSGT ORFs was confirmed by sequencing with the pGEX 5′ primer (Arró et al., 2014). To express the GST-SlSGT fusion proteins, overnight cultures of the transformed *E. coli* cells (3 mL) were grown at 37°C in LB medium supplemented with ampicillin (100 mg/L) and kanamycin (25 mg/L). The overnight cultures were used to inoculate 100 mL of fresh LB supplemented with the same concentrations of ampicillin and kanamycin. Cultures were grown at 37°C until an OD_600_ of 0.5–0.6 and expression of the recombinant proteins was then induced by the addition of isopropyl-1-thio-β-D-galactopyranoside (IPTG) to a final concentration of 0.1 mM. The IPTG-induced cultures were grown overnight at room temperature and cells were harvested by centrifugation at 7,000 × *g* at 4°C for 5 min. Cell pellets were washed with distilled water, resuspended in 10 mL of reaction buffer (100 mM Tris-HCl pH 8.0, 10 mM MgCl_2_, 1 mM EDTA, 1 mM DTT and 0.2% (v/v) Triton X-100), and disrupted by sonication for 3 min at 30 s intervals while being chilled in a -10°C bath. The cell lysates were centrifuged at 15,000 × *g* for 30 min at 4°C to pellet cell debris and the soluble protein extracts were collected and assayed for SGT enzyme activity.

### SGT Enzyme Activity Assay

The SGT activity assays were carried out in a final volume of 200 μL containing 150 μL of *E. coli* soluble protein extract, 20 μL of a plant sterol mixture (25 mg/mL) consisting of approximately 13% brassicasterol, 26% campesterol, 7% stigmasterol, and 53% β-sitosterol (Matreya) in ethanol, and 20 μL of 3.6 mM UDP-glucose (Calbiochem). After incubation at 30°C for 2 h, the reaction was stopped by adding 0.8 mL of a 0.45% (w/v) NaCl solution. The sterol fraction was then extracted with 4 mL of a chloroform/methanol mixture (2:1), which was subsequently evaporated to dryness. The dried residue was dissolved in 100 μL of chloroform/methanol mixture (2:1) and FS and SG were separated by thin layer chromatography (TLC) using precoated silica gel PLC 60 F254 plates (20 cm × 20 cm) (Merck, Darmstadt) and dichloromethane/methanol (9:1) as a mobile phase. Free cholestanol and cholestanyl-β-D-glucoside standards were also applied onto the TLC plates. Plates were sprayed with a 0.01% primuline (Sigma-Aldrich) solution and FS and SG bands were detected with an UV lamp. The SG bands were scraped from the silica plates and 1.5 mL of a 2 N HCl methanolic solution was added to the silica powder. After incubation at 85°C for 2 h to hydrolyze SG, the reaction was quenched with 1.5 ml of 0.9% (w/v) NaCl and the FS moieties were extracted twice with 3 mL of *n*-hexane. The hexanic phases were collected by centrifugation, mixed and evaporated to dryness. Sterols were derivatized by adding 50 μL of Bis(trimethylsilyl) trifluoroacetamide (BSTFA) (Regis technologies) followed by incubation for 2 h at 80°C. The sterol samples were then evaporated to dryness, dissolved in 100 μL of isooctane and analyzed using gas chromatography-mass spectrometry (GC-MS). GC-MS analyses were performed using an Agilent 7890A gas chromatograph equipped with a Sapiens-X5ms capillary column (30 m × 0.25 mm × 0.25 μm) (Teknokroma) and coupled with a 5975C mass spectrometer (Agilent).

### RT-qPCR Analysis of *SlSGT* Gene Expression

The cDNA samples for RT-qPCR gene expression analysis were prepared from DNA-free total RNA samples obtained as indicated above. Real-time PCR reactions were performed with a LightCycler 480 equipment (Roche Diagnostics) in a total volume of 20 μl containing 10 μl LightCycler 480 SYBR Green I Master (Roche Diagnostics), 0.6 μl forward primer (0.3 μM), 0.6 μl reverse primer (0.3 μM), 6.8 μl water and 2 μl cDNA (50 ng). The LightCycler experimental run protocol used was: 95°C for 10 min followed by 40 cycles of 95°C for 10 s, 60°C for 30 s and a final cooling step to 4°C. The raw PCR data from LightCycler software 1.5.0 were used in the analysis. Specific primer pairs for *SlSGT* mRNAs and the tomato Clathrin Adaptor Complexes medium subunit (CAC) gene (Solyc08g006960) used as a housekeeping reference gene are described in Supplementary Table S2. For efficiency determination, a standard curve of six serial dilution points (ranging from 6.25 to 200 ng) was made in triplicate. Amplification efficiencies of target and reference genes were almost equal. Dissociation curves for each PCR product were examined for non-specific amplification. Quantification of transcript levels was done in three independent biological replicates, and for each biological replicate three technical replicates were performed. The cycle threshold change (DCT) was calculated as follows: DCT = CT (Target) - CT (CAC), and the fold change value was calculated using the 2^-ΔCT^ expression (Livak and Schmittgen, 2001).

### High-Throughput RT-qPCR Analysis of *SlSGT* Gene Expression in Response to Different Effectors

Tomato seedlings grown in MS liquid medium and treated as described above were sampled at different time points (0, 3, 6, 12, 24, and 48 h) and RNA was extracted with a Maxwell^®^ 16 LEV Plant RNA kit (Promega) using a Maxwell 16 Instrument (Promega) according to manufacturer’s instructions. *SlSGT* gene expression was quantified by real-time PCR using the Biomark^TM^ instrument (Fluidigm corporation, San Francisco, CA, United States) and 2× SsoFast^TM^ EvaGreen^®^ Supermix with low Rox (Bio-Rad^1^) dye. The synthesis of cDNA was performed as described above for standard RT-qPCR and the cDNA samples (approximately 50 ng/μL^-1^) were pre-amplified using TaqMan PreAmp Master Mix (Applied Biosystems, Lifetechnologies) and then diluted to 1:10. Primers (Supplementary Table S2) were used at a final concentration of 500 nM. After pre-amplification, cDNAs were treated with exonuclease I to remove leftover primers. The PCR efficiency for each primer pair was calculated according to a dilution series from a pooled cDNA sample including all biological treatments. Quantification of transcript levels was done in three independent biological replicates and for each biological replicate two technical replicates were performed. Relative expression was calculated using Data Analysis Gene (DAG) Expression software^2^ (Ballester et al., 2013) through the construction of standard curves for relative quantification and reference genes for sample normalization. Eight reference genes were included in the expression analysis but after assessing their expression stability under the different treatments, only *PP2Acs* (Solyc02g093800.2.1) and *EF1a* (SGN-U590849) were used to normalize *SlSGT* gene expression data. Statistical significance of changes in *SlSGT* transcript levels was calculated by using unpaired *t*-tests.

### Subcellular Localization of SlSGT Proteins

The SlSGT coding sequences lacking the stop codon were amplified using pENTR-SlSGT plasmids as template, specific primer pairs (Supplementary Table S1) and AccuPrime Taq DNA polymerase. The PCR products were cloned into pENTR/D-TOPO vector and the verified sequences were subcloned by Gateway recombination into the binary vector pEarley-Gate101 (Earley et al., 2006), obtained from the Arabidopsis Biological Resource Centre (ABRC)^3^ (stock CD3-683), to generate YFP fusions at the C-terminus of the SlSGT proteins. The Arabidopsis UGT80A2 and UGT80B1 coding sequences without stop codon in plasmids PDONR_AT3G07020.2 and PDONR_AT1G43620.1, respectively, were also obtained from ABRC stocks and subcloned into pEarley-Gate101 plasmid in order to generate the corresponding YFP fusions at the C-terminus of the SGT proteins. All constructs were sequenced to confirm the in-frame fusions. In all cases the coding sequences were under the control of the *CaMV35S* gene promoter. Recombinant plasmids coding for the tomato and Arabidopsis SGT-YFP fusions were transformed by electroporation into *Agrobacterium tumefaciens* strain C58C1 (pGv2260) (Deblaere et al., 1985). The resulting strains were separately mixed in a 1:1 ratio with an *Agrobacterium tumefaciens* strain harboring the HC-Pro silencing suppressor (Goytia et al., 2006) and infiltrated in leaves of 3- to 5-week-old *N. benthamiana* plants employing the syringe infiltration method. Plants were kept growing under long-day conditions at 25°C and after 3 days leaves were also infiltrated with a propidium iodide (PI) solution (5 mg/mL) to stain the cell wall. Pieces of the agroinfiltrated leaves were then collected for confocal laser scanning microscopy analysis. The abaxial epidermis of agroinfiltrated leaf tissue was scanned with an Olympus FV1000 confocal microscope (Tokyo, Japan) using the 60x water-immersion NA: 1.20 objective. The 488 nm argon laser was used to excite the YFP and the 599 nm diode laser was used for PI excitation. The emission windows for visualization of fluorescence were set at 500–545 nm and 570–670 nm, respectively. FV10-ASW software (Olympus) was used for image capture and ImageJ-32^4^ for merging false-colored images of transiently co-transformed cells. For fluorescence recovery after photobleaching (FRAP) analysis, the abaxial side of the agroinfiltrated leaf fragments was scanned using the microscope settings described above. A 7–10 μm region of interest (ROI) was defined and photobleached using full laser power (100%) for 1 s. To assess the recovery of fluorescence the entire focused cell area was monitored with a low laser power (15%) during 60 s. The image previous to the bleaching was acquired with the same laser power. The obtained data were normalized as previously described (Luu et al., 2012), and a two-phase exponential equation was used to model the normalized data. GraphPad software (GraphPad Software Inc.) was used for FRAP curves fitting.

For tissue fractionation into membrane and soluble fractions, approximately 3 g of *N. benthamiana* agroinfiltrated leaf zones were harvested from three independent plants, cut in small pieces and quickly mixed with 20 mL of ice-cold lysis buffer [0.3 M sucrose, 50 mM 3-(-*N*-morpholino) propanesulfonic acid (MOPS)-NaOH, (pH 7.5) and 5 mM EDTA], supplemented immediately before use with 0.5% (w/v) polyvinylpirrolidone, 5 mM DTT, 5 mM ascorbic acid and a mixture of protease inhibitors (10 mM leupeptin, 1 mM pepsatin, 10 mM E64, 0,3 mM aprotinin and 1 mM PMSF). Leaf tissue was homogenized with an Ultra Turrax homogenizer (3× 30 s at medium speed on ice) and the resulting homogenate was filtered through two layers of nylon cloth. PMSF (100 mM stock solution) was added to the filtered homogenate to get 1 mM final concentration before centrifugation at 10,000 × *g* for 15 min at 4°C to remove cell debris. The resulting supernatant was recovered and centrifuged again at 10,000 × *g* for 15 min at 4°C. The pellet was discarded and the supernatant was centrifuged at 100,000 × *g* for 60 min at 4°C to obtain a pellet (P100; membrane fraction) and a supernatant (S100; soluble fraction). The P100 was then resuspended in 10 ml of fresh resuspension buffer [0.3 M sucrose, 5 mM sodium phosphate (pH 7.8), 0.1 mM EDTA, 1 mM DTT and 1 mM PMSF] and both the S100 and the washed pellet were centrifuged again at 100,000 × *g* for 60 min at 4°C. The resulting P100 and S100 fractions were processed once again as described above to obtain the final P100 and S100 fractions. The P100 pellet was subsequently resuspended in 1 mL of resuspension buffer for immunoblot analysis. To strip the P100 pellets with salt and urea, washed pellets (final P100 fraction) were resuspended in 8 mL of resuspension buffer supplemented with either 1 M NaCl or 8 M urea and, after incubation at 4°C for 30 min with constant shaking, centrifuged at 100,000 × *g* for 60 min at 4°C to obtain the corresponding membrane and soluble fractions. Pellets were then resuspended in 1 mL of resuspension buffer to obtain the final NaCl- and urea-stripped membrane fractions. For immunoblot analysis, equivalent amounts of P100 (1 to 3 μg of protein) and S100 fractions (15 to 20 μg of protein) from each *N. benthamiana* leaf sample were fractionated by 10% SDS–PAGE (Laemmli, 1970), transferred to a nitrocellulose membrane (Amersham, GE Healthcare) and probed using a rabbit anti-GFP antibody (Invitrogen) at a 1:1000 dilution. Secondary donkey anti-rabbit IgG conjugated to horseradish peroxidase was used at a 1:10000 dilution. The protein-YFP antibody complexes were visualized using the Amersham ECL Select Western Blotting Detection Reagent (GE Healthcare) according to the manufacturer’s instructions and the ChemiDoc Touch (Bio-Rad) for chemiluminescence detection.

### Sterol Analysis

For SG determination, approximately 2 g of the *N. benthamiana* agroinfiltrated leaf zones from the same plants used for tissue fractionation studies were collected and frozen in liquid nitrogen, grinded to a fine powder and lyophilized. Thirty micrograms of the lyophilized powder were placed in a glass tube and 100 μl of 0.1 mg/mL cholestanyl-β-D-glucoside in chloroform/methanol (2:1) were added as an internal standard. The sterol fraction was then extracted with 3 mL of a chloroform–methanol solution (2:1). After vigorous vortexing and sonication for 10 min at room temperature, 1.5 mL of 0.9% (w/v) NaCl were added to facilitate further phase separation. The organic phase was recovered by centrifugation at 5,000 × *g* for 5 min at room temperature and transferred to a new tube. The remaining aqueous phase was extracted again with 3 mL of the chloroform–methanol mixture (2:1) and the two organic extracts were mixed together and subsequently evaporated to dryness. The dried residue was dissolved in 100 μL of chloroform/methanol (2:1) and the SG fraction was purified by TLC and quantified by GC-MS as described above.

## Results

### Identification and Cloning of Candidate Genes Encoding Tomato UDP-Glucose:Sterol Glycosyltransferases

**FIGURE 2:**
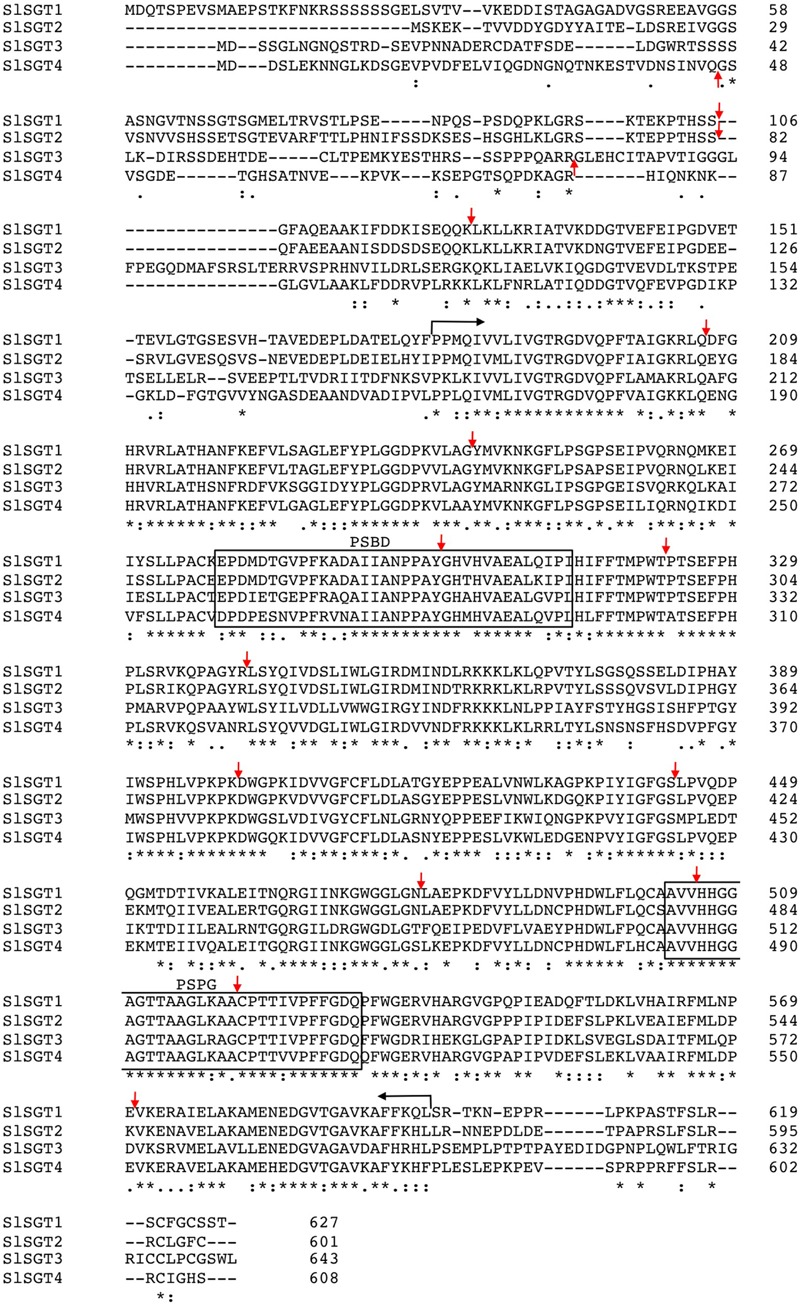
Multiple sequence alignment of *Solanum lycopersicum* cv. Micro-Tom SGT proteins. Amino acid sequences were aligned using the Clustal Omega multiple sequence alignment tool (http://www.ebi.ac.uk/Tools/msa/clustalo/). Amino acid residues are numbered on the right. Asterisks denote residues conserved in all four sequences. Colons indicate conservation between amino acid groups of strongly similar properties whereas periods indicate conservation between amino acid groups of weakly similar properties. Hyphens indicate gaps introduced to optimize the alignment. Vertical arrows denote positions at which introns interrupt the SlSGT amino acid sequences, whereas horizontal arrows delimit a highly conserved core region of 420 amino acids in length that contains the putative steroid-binding domain (PSBD) found in steroid UDP-glucuronosyltransferases and a plant secondary product glucosyltransferase (PSPG) signature sequence suggested to represent an UDP-sugar binding domain, which are boxed. The sequences shown have the following GenBank accession numbers: KY354517 (SlSGT1), KY354518 (SlSGT2), KY354519 (SlSGT3), and KY354520 (SlSGT4).

**FIGURE 3:**
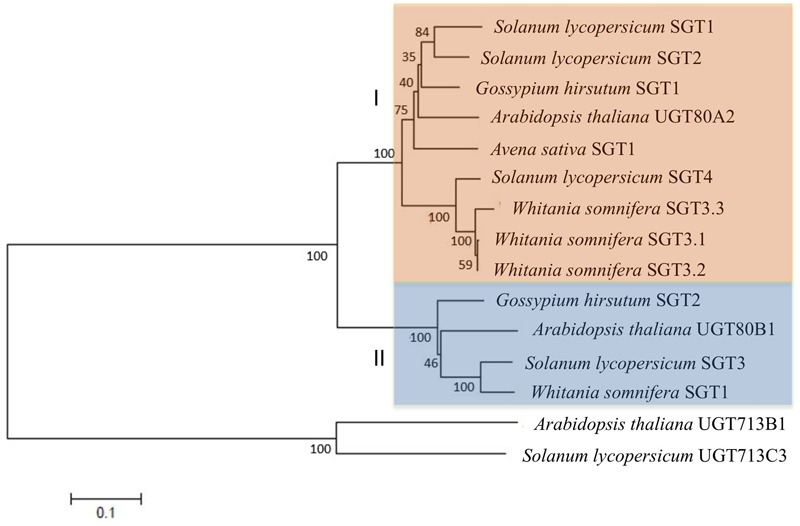
Phylogenetic tree showing the relatedness of SlSGT (*Solanum lycopersicum*) proteins with previously reported functional plant SGTs. Phylogenetic analysis was performed using the neighbor-joining method and the following SGT protein sequences obtained from NCBI database: *Arabidopsis thaliana* UGT80A2 (Z83833) and UGT80B1 (BT005834); *Withania somnifera* SGT1 (DQ356887), SGT3.1 (EU342379), SGT3.2 (EU342374), and SGT3.3 (EU342375); *Gossypium hirsutum* SGT1 (KJ572778) and SGT2 (KJ572779) and *Avena sativa* SGT1 (Z83832). The percentage of replicate trees in which the associated taxa clustered together in the bootstrap test (1000 replicates) is shown. Analyses were conducted using MEGA version 7 (Kumar et al., 2016). UDP-glucosyltransferases UGT713B1 (NP_568452), from *Arabidopsis thaliana*, and UGT713C3 (XP_004230516.1), from *Solanum lycopersicum* were used as outgroup.

In order to identify tomato UDP-glucose:sterol glycosyltransferase candidates, we performed a search in the Phytozome database^5^ using as query the amino acid sequence of the Arabidopsis SGTs, UGT80A2 and UGT80B1 (DeBolt et al., 2009). This analysis enabled the identification of four putative SGT proteins, which will be further referred to as SlSGT1 (Solyc06g007980.2.1), SlSGT2 (Solyc09g061860.2.1), SlSGT3 (Solyc04g071540.2.1) and SlSGT4 (Solyc04g051150.2.1). Next we amplified the corresponding ORFs by PCR using as a template cDNA synthesized from RNA obtained from tomato (*S. lycopersicum* cv. Micro-Tom) pericarp tissue (SlSGT1, SlSGT2, and SlSGT3) or seedlings treated with ABA (SlSGT4) and specific primer pairs (Supplementary Table S1). Alignment of the cloned cDNAs with the corresponding genomic sequences in the Phytozome database revealed that *SlSGT* genes share the same exon–intron organization, consisting of 14 exons separated by 13 introns that are located at equivalent positions in the four genes (**Figure 2**). Conceptual translation of the cDNA sequences showed that the encoded proteins consist of 627 (SlSGT1), 601 (SlSGT2), 643 (SlSGT3), and 608 (SlSGT4) amino acids (**Figure 2**), which are identical to the amino acid sequences found in the Phytozome database and share among them overall identity values ranging from 50 to 80% (similarity ranging from 64 to 85%) (Supplementary Table S3). SlSGT sequence conservation is largely concentrated in a central region of 420 amino acids in length that includes a putative steroid-binding domain (PSBD) found in steroid UDP-glucuronosyltransferases and a C-terminal plant secondary product glucosyltransferase (PSPG) signature sequence characteristic for a superfamily of nucleoside diphosphosugar glycosyltransferases suggested to represent an UDP-sugar binding domain. By contrast, SlSGT proteins show almost no sequence conservation within a large N-terminal region ranging from 155 (SlSGT2) to 180 (SlSGT1) amino acids in length and a much shorter C-terminal region ranging from 27 (SlSGT2) to 41 (SlSGT3) amino acids (**Figure 2**). Protein sequence alignments of the four SlSGTs with other functional plant SGTs (Supplementary Figure S1) revealed also a broad range of identity values, from 51% (SlSGT4 vs. UGT80B1) to 91% (SlSGT4 vs. WsSGT3.3) (Supplementary Table S3). Comparison of these sequences in a neighbor-joining phylogeny (**Figure 3**) identified two main clades (I and II) including tomato SGT proteins. The closely related SlSGT1 and SlSGT2 proteins (80% identical) form a subgroup within the clade I, which also includes SGTs from *A. thaliana* UGT80A2, *G. hirsutum* SGT1 and *A. sativa* SGT1. SlSGT4 falls in a separate subgroup within the same clade, together with the *W. somnifera* SGT3.1, SGT3.2, and SGT3.3 proteins. While, SlSGT3 was found in the second main clade (II), which also includes SGT1 from *W. somnifera*, UGT80B1 from *A. thaliana* and SGT2 from *G. hirsutum*. Altogether, the above sequence analysis strongly suggested that the four tomato SGT candidates were active forms of the enzyme.

### Functional Characterization of Tomato SGTs Expressed in *E. coli*

**FIGURE 4:**
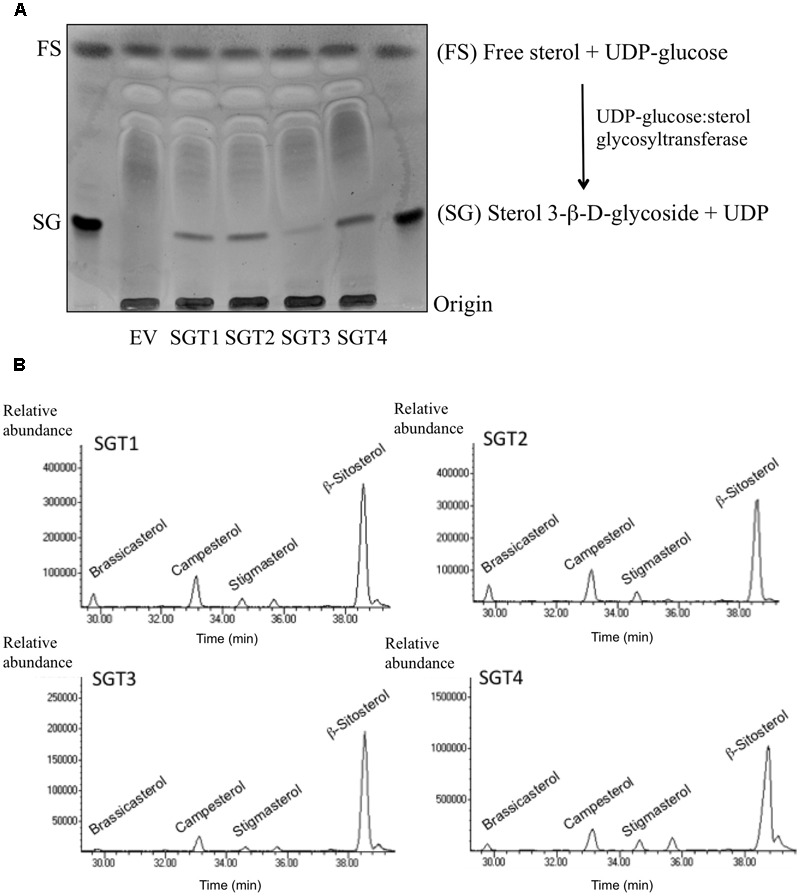
Functional characterization of *S. lycopersicum* cv. Micro-Tom SGT isozymes. **(A)** SGT enzyme activity assays were performed by incubating the soluble fraction of extracts from *E. coli* cells harboring empty vector pGEX-3X-NotI (EV) or recombinant plasmids pGEX-3X-NotI-SGT, enabling expression of the four tomato GST-SGT fusion proteins (SGT1 to 4), with a mixture of plant sterols and UDP-glucose as substrates. The reaction products were separated by normal-phase TLC. The position of cholestanol (FS) and cholestanyl glycoside (SG) standards is shown on the left. A simplified scheme of the reaction catalyzed by SGT (UDP-glucose:sterol glycosyltransferase) is shown on the right. **(B)** GC chromatograms of the FS recovered by acid hydrolysis from the SG bands scraped off the TLC plates showing the peaks corresponding to the four sterols present in the FS mixture used as a substrate.

In order to verify that the cloned cDNAs actually encode functional SGTs, the corresponding ORFs were cloned in frame downstream of the glutathione *S*-transferase (GST) coding sequence into pGEX-3X-NotI expression vector (Arró et al., 2014). The resulting recombinant plasmids were introduced into *E. coli* cells and expression of the GST-SlSGT fusion proteins was induced with IPTG. The cell-free soluble fraction of bacterial lysates from cells expressing the GST-SlSGT fusions was assayed for SGT activity using a mixture of brassicasterol, campesterol, stigmasterol, and β-sitosterol as sterol acceptor and UDP-glucose as a sugar donor. The reaction products were then fractionated by TLC and stained with primuline for sterol visualization. As expected, a primuline-stained band with the same mobility than the cholestanyl glucoside, used as a standard for the glycosylated products, was visible in extracts from *E. coli* cells expressing the recombinant proteins but not in extracts from cells harboring the empty pGEX-3X-NotI vector (**Figure 4A**). Analysis by GC-MS of the putative SG bands scraped from the TLC plates revealed that the four SlSGTs were able to glycosylate all sterol species present in the mixture (**Figure 4B**), thus confirming that the proteins encoded by the *SlSGT* genes actually display SGT activity and therefore are the true tomato orthologs of Arabidopsis UGT80A2 and UGT80B1.

### Expression Analysis of *SlSGT* Genes

**FIGURE 5:**
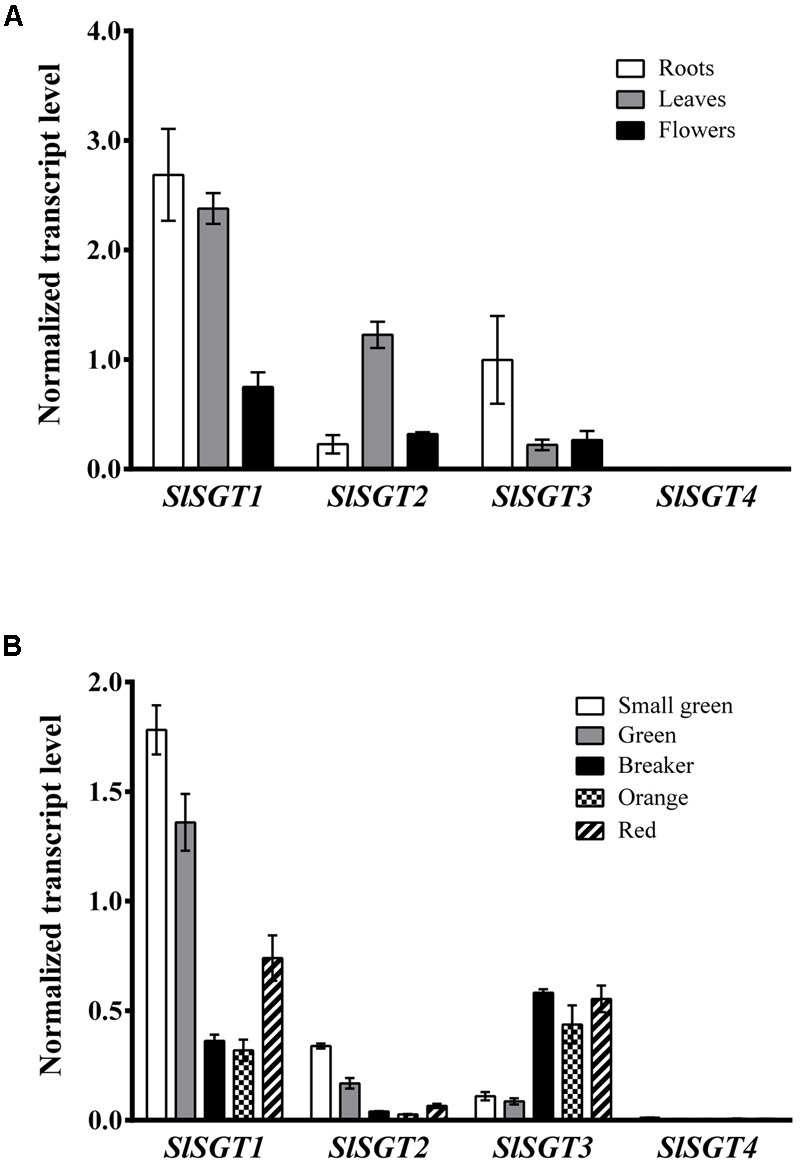
RT-qPCR analysis of SlSGT mRNA levels in RNA samples from tomato **(A)** root, leaf, and flower tissue and **(B)** fruits at the indicated developmental and ripening stages. Transcript levels were normalized relative to the mRNA levels of the Clathrin Adaptor Complexes medium subunit (CAC) gene (Solyc08g006960). Values are means ± SD (*n* = 9).

We next performed RT-qPCR analyses to investigate the expression of the *SlSGT* genes in different organs of tomato plants and fruits at different stages of development and ripening (**Figure 5**). Among the four *SlSGT* genes, *SlSGT1* was the most intensely expressed in root, leaf, and flower tissues, being roots and leaves the tissues where the highest levels of SlSGT1 mRNA were detected. Leaves and roots were also the tissues where the highest levels of SlSGT2 and SlSGT3 mRNAs were found, respectively, though in both cases mRNA levels were lower than those of SlSGT1 (**Figure 5A**). Analysis of SlSGT mRNA levels in fruits (**Figure 5B**) revealed that *SlSGT1* was again the most actively expressed *SlSGT* gene in both developing (small green and mature green stages) and mature red fruits. Interestingly, SlSGT1 mRNA levels decreased when fruits started to ripe (breaker and orange stages) but increased again in red ripe fruits. The qualitative profile of *SlSGT2* expression was very similar, though in all tested samples the SlSGT2 mRNA levels were lower than those of SlSGT1. On the contrary, the expression pattern of *SlSGT3* was clearly different since its mRNA levels were low in developing fruits (small green and mature green stages) but increased sharply when fruits started to ripe and remained at similar high levels until the red mature stage. Interestingly, *SlSGT4* transcripts were barely detectable in all samples analyzed. Comparison of the RT-qPCR expression results obtained in this study (**Figure 5**) with the RNA-seq expression data for the *SlSGT* genes of *S. lycopersicum* cv. Heinz available via the tomato eFP browser at bar.utoronto.ca (Supplementary Figure S2) showed a high degree of qualitative and quantitative consistency despite *SlSGT* expression data were obtained from different tomato varieties (Micro-Tom vs. Heinz) using different analytical methodologies (RT-qPCR vs. RNA-seq). Overall, the results from these expression analyses demonstrated that the *SlSGT* genes are differentially expressed in different organs of tomato plants and also throughout the different stages of fruit development and ripening.

### Subcellular Localization of SlSGT Proteins

**FIGURE 6:**
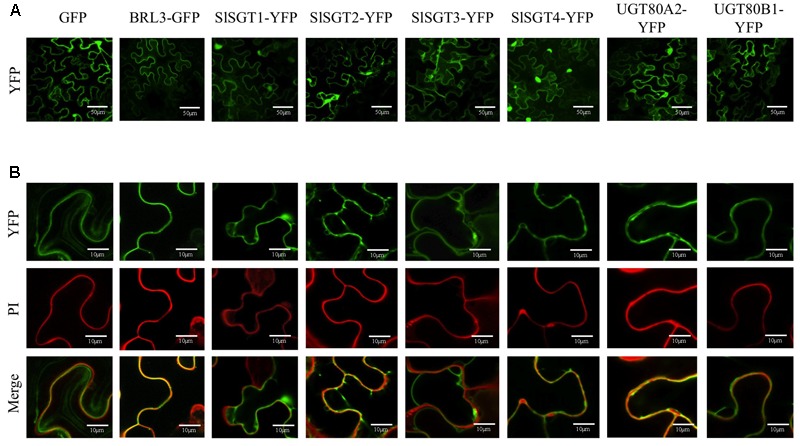
**(A)** Confocal optical sections showing the YFP fluorescence pattern of *N. benthamiana* cells expressing SGT(1-4)-YFP, UGT80A2-YFP UGT80B1-YFP, and BRL3-GFP protein fusions as well as free GFP. Cells were also stained with propidium iodide (IP) to mark the cell wall. Bars = 50 mM. **(B)** Close-up view of selected regions of the above cells showing fluorescence of YFP (upper images) and IP (middle images), and the corresponding merged images (bottom images). Bars = 10 mM.

**FIGURE 7:**
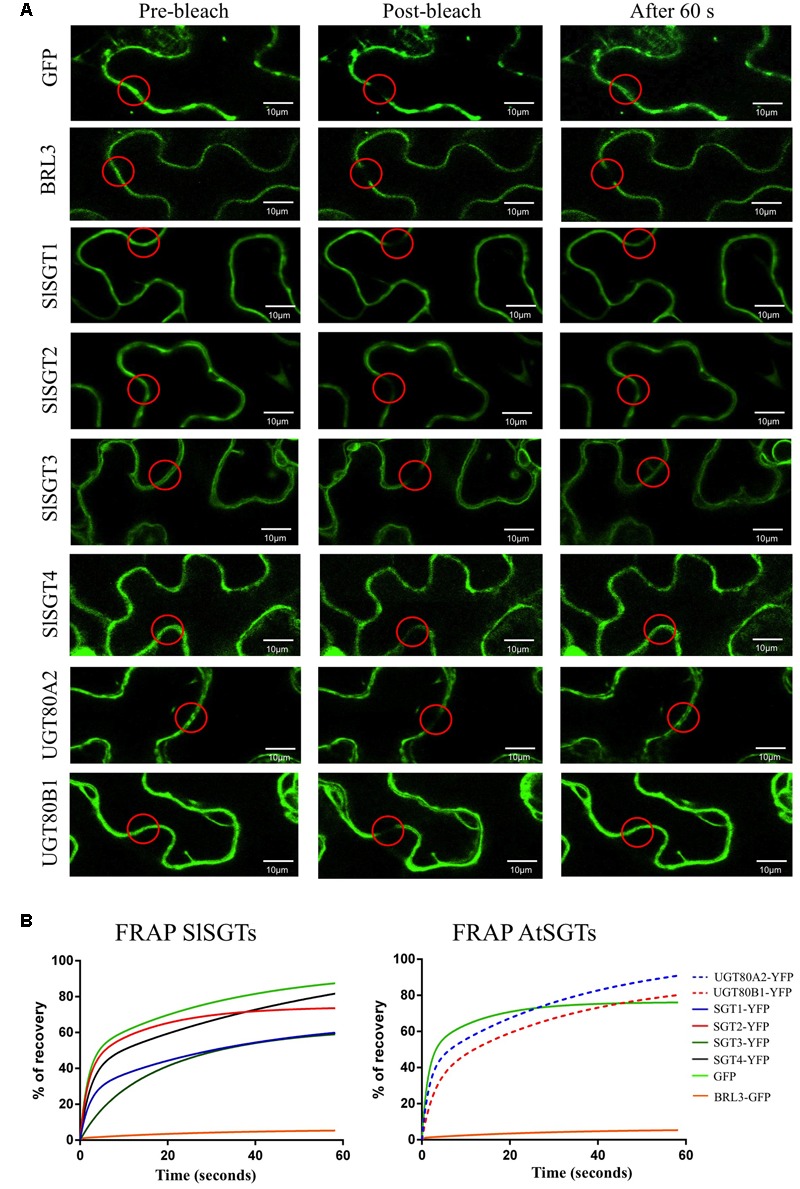
**(A)** FRAP analysis in *N. benthamiana* cells transiently expressing tomato and Arabidopsis SGTs fused C-terminally to YFP. GFP and BRL3-GFP proteins were also transiently expressed as cytosolic and plasma membrane control proteins, respectively. Regions of interest (ROI) in cells expressing the fluorescent proteins (red circles) were photobleached with a pulse (1 s) of high intensity laser light. The images shown were acquired just before photobleaching (left column), immediately after photobleaching (middle column) and after 60 s of fluorescence recovery (right column). **(B)** FRAP curves representing the time course of SlSGT(1 to 4)-YFP, UGT80A2-YFP, UGT80B1-YFP, BRL3-GFP, and GFP fluorescence recovery between time points 0 and 60 s. Fluorescence recovery at the different time points is expressed as percentage of fluorescence at time point 0 s (pre-bleach). Fluorescence recovery curves represent the best fits from normalized datasets of 20 independently bleached ROIs (Supplementary Figure S3).

**FIGURE 8:**
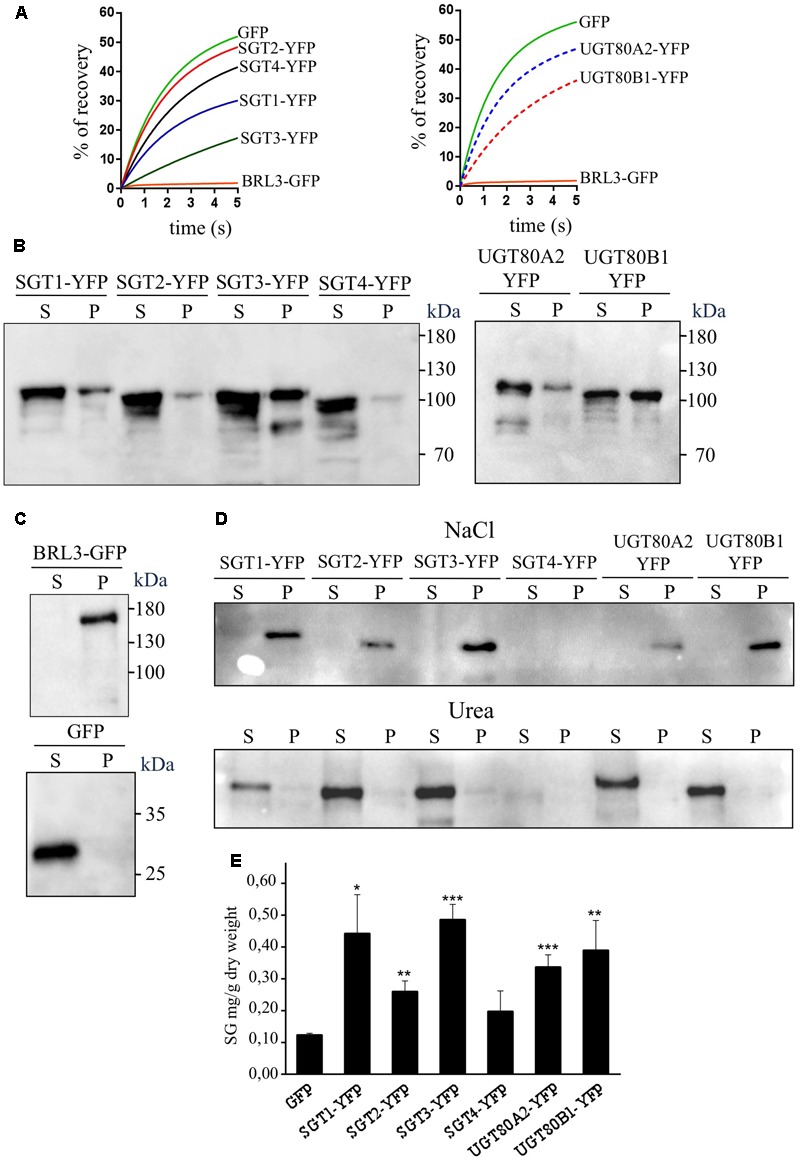
**(A)** Close-up view of the FRAP curves shown in **Figure 7** representing the time course of SlSGT(1 to 4)-YFP, UGT80A2-YFP, UGT80B1-YFP, BRL3-GFP, and GFP fluorescence recovery between time points 0 and 5 s. **(B)** Immunoblot analysis of soluble (S) and membrane (P) cell fractions obtained from agroinfiltrated *N. benthamiana* leaf zones expressing tomato SGT-YFP fusion proteins (SGT1 to 4) and the Arabidopsis UGT80A2-YFP and UGT80B1-YFP proteins. **(C)** Soluble and membrane cell fractions from leaves expressing BRL3-GFP and GFP proteins were also analyzed as controls of membrane- and cytosol-localized proteins, respectively. The predicted molecular weight of fusion proteins is approximately 94.0 kDa (SlSGT1-YFP), 92.5 kDa (SlSGT2-YFP), 97.5 kDa (SlSGT3-YFP), 93.0 kDa (SlSGT4-YFP), 95.5 kDa (UGT80A2-YFP), 94.5 kDa (UGT80B1-YFP), and 153.0 kDa (BRL3-GFP). The position of protein molecular-weight standards is shown on the right. **(D)** Immunoblot analysis of membrane fractions stripped with 1 M NaCl and 8 M urea (P), and the corresponding soluble fractions (S). **(E)** Sterol glycoside content in agroinfiltrated *N. benthamiana* leaf zones transiently expressing SlSGT(1 to 4)-YFP, UGT80A2-YFP, UGT80B1-YFP, and GFP. Tissue samples were collected 3 days after agroinfiltration and SG levels were determined as described in “Materials and Methods” Section. Total SG content includes glycosylated cholesterol, campesterol, stigmasterol, and β-sitosterol. Values are means ± SD (*n* = 3). Asterisks show the values that are significantly different (^∗^*p* < 0.05, ^∗∗^*p* < 0.01, ^∗∗∗^*p* < 0.001) compared to those in leaf samples expressing GFP.

The analysis of the primary structure of SlSGTs with the TMHMM 2.0 program (Krogh et al., 2001) predicted that none of the tomato SGTs contains amino acid sequences meeting the requirements to serve as true transmembrane domains. A similar result was obtained when the Arabidopsis UGT80A2 and UGT80B1 proteins were submitted to the same analysis. Furthermore, none of the above SGT proteins is predicted to have a sequence potentially serving as a signal peptide according to the results obtained with the bioinformatic tool TargetP (Emanuelsson et al., 2007). All these observations suggested that tomato and Arabidopsis SGTs do not localize in the PM. This, together with the fact that there is no clear consensus about the subcellular localization of plant SGTs, prompted us to determine experimentally the subcellular localization of tomato and Arabidopsis SGTs. To this end, we transiently expressed the corresponding C-terminal fusions of SGTs with the yellow fluorescent protein (YFP) in *N. benthamiana* leaves and analyzed the resulting fluorescence pattern by confocal laser microscopy. To delimit the cell membrane, *N. benthamiana* cells expressing the above fluorescent proteins were also stained with PI prior to confocal analysis. As shown in **Figure 6A**, the YFP signal of the SlSGTs-YFP, UGT80A2-YFP, and UGT80B1-YFP fusions appeared as a thick band at the cell periphery similar to that observed in cells expressing GFP and the brassinosteroid receptor BRL3 fused to the GFP (BRL3-GFP) (Caño-Delgado et al., 2004), which were also expressed as controls for cytosolic and PM localization, respectively. As can be observed in **Figure 6B**, PI and BRL3-GFP fluorescence patterns clearly overlapped, which was not the case when the PI signal was compared to that of GFP, SlSGTs-YFP, UGT80A2-YFP, and UGT80B1-YFP, thus leaving still unresolved the question of whether tomato and Arabidopsis SGT proteins localize to the cytosol or the PM. In epidermal cells of *N. benthamiana* leaves the cell vacuole compresses the cytoplasm and its content against the PM and the rigid cell wall, which makes difficult to distinguish between fluorescence signals of PM and cytosolic proteins. To clarify this issue, we performed FRAP analyses (**Figure 7**), a technique that allows evaluation of the rate of protein mobility in living cells (Reits and Neefjes, 2001; Wu et al., 2006; Held et al., 2008; Ishikawa-Ankerhold et al., 2012). To this end, selected regions in cells expressing the above fluorescent proteins were irradiated with a short pulse of high intensity laser light to produce an irreversible photobleaching of the fluorophore in the ROI (**Figure 7A**). Then, the recovery of fluorescence in the irradiated area due to the migration of the non-photobleached fusion proteins back to the bleached area was monitored at different time points over a period of 60 s (**Figure 7B** and Supplementary Figure S3). The rate and percentage of fluorescence recovery due to the exchange between photobleached and intact fluorescent protein molecules depends on the mobility of the fluorescent protein. Free cytosolic proteins can easily move and therefore show high fluorescence recovery rates contrary to membrane proteins, which hardly can be replenished in the bleached area. As shown in **Figure 7B**, after a short pulse (1 s) of laser light, the rate of fluorescence recovery in cells expressing SlSGT2-YFP and SlSGT4-YFP was similar to that observed in cells expressing cytosolic GFP. By contrast, fluorescence recovery in cells expressing SlSGT1-YFP and SlSGT3-YFP was slower compared to GFP. In both cases less than 60% of initial fluorescence intensity was recovered after 60 s while in the GFP control fluorescence recovery was near the 90% at the same time point. These differences became more evident when FRAP profiles over the first 5 s after photobleaching were compared (**Figure 8A**). Fluorescence values at this time point were 33% (SlSGT3-YFP), 60% (SlSGT1-YFP), 80% (SlSGT4-YFP), and 94% (SlSGT2-YFP) of that in cells expressing GFP. These results are in sharp contrast to that obtained in bleached areas of cells expressing the PM protein BRL3-GFP where, as expected, no significant recovery of fluorescence could be observed over the monitored period of time (**Figures 7B, 8A** and Supplementary Figure S3). Interestingly, a differential FRAP response was also observed in the case of Arabidopsis UGT80A2-YFP and UGT80B1-YFP since recovery values at 5 s after photobleaching were 62% (UGT80B1-YFP) and 88% (UGT80A2-YFP) of that in cells expressing GFP (**Figure 8A**). Overall these observations suggested that SlSGT1-YFP, SlSGT3-YFP, and UGT80B1 cannot move as freely as GFP to replenish the bleached region, while SlSGT2-YFP, SlSGT4-YFP, and UGT80A2 behave much like GFP.

To further investigate the subcellular localization of SGTs, we also performed immunoblot analysis of membrane and soluble fractions obtained from agroinfiltrated *N. benthamiana* leaves expressing SlSGTs-YFP, UGT80A2-YFP, UGT80B1-YFP, BRL3-GFP, and GFP (**Figures 8B,C**). Interestingly, tomato SGT2-YFP and SGT4-YFP, as well as Arabidopsis UGT80A2-YFP, were primarily detected in the soluble fraction. SlSGT1-YFP also predominated in the soluble fraction although the proportion of protein detected in the membrane fraction was higher than that of SlSGT2-YFP and SlSGT4-YFP. By contrast, tomato SGT3-YFP and Arabidopsis UGT80B1-YFP proteins were roughly equally distributed between the membrane and soluble fractions. As expected, GFP and BRL3-GFP were only detected in the soluble and membrane fractions, respectively (**Figure 8C**), indicating that cross-contamination between fractions, if any, was negligible. Altogether these results were fully consistent with those obtained in the FRAP analysis. Moreover, our finding that overexpression of tomato and Arabidopsis SGT-YFP proteins in *N. benthamiana* resulted in increased levels of SGs (**Figure 8E**) demonstrated that the overexpressed proteins are active in a native environment, thus providing further support to the view that under our experimental conditions immunoblot analysis truly reflects the observed differential distribution of tomato and Arabidopsis SGTs between the cytosol and the PM. To assess the tightness of association of the different SGT-YFP fusion proteins with the PM, the above membrane fractions were subsequently treated with either 1 M NaCl or 8 M urea (**Figure 8D**). Treatment with a high ionic strength buffer did not solubilize any of the membrane-bound SGT-YFP proteins. On the contrary, SGT-YFP proteins were recovered in the soluble fraction when membrane fractions were treated with urea, a chaotropic agent. It has to be mentioned that SlSGT4-YFP was almost undetectable in these fractions due to the very low levels of this protein present in the untreated membrane fraction (**Figure 8B**). Altogether, these observations suggest that SGT-YFP proteins detected in the membrane fraction are loosely associated with the PM, likely through hydrophobic interactions, and thereby should be considered as peripheral membrane proteins, which is, moreover, fully consistent with the predicted lack of transmembrane domains.

### Transcriptional Profiling of *SlSGT* Gene Expression in Response to Stress

**FIGURE 9:**
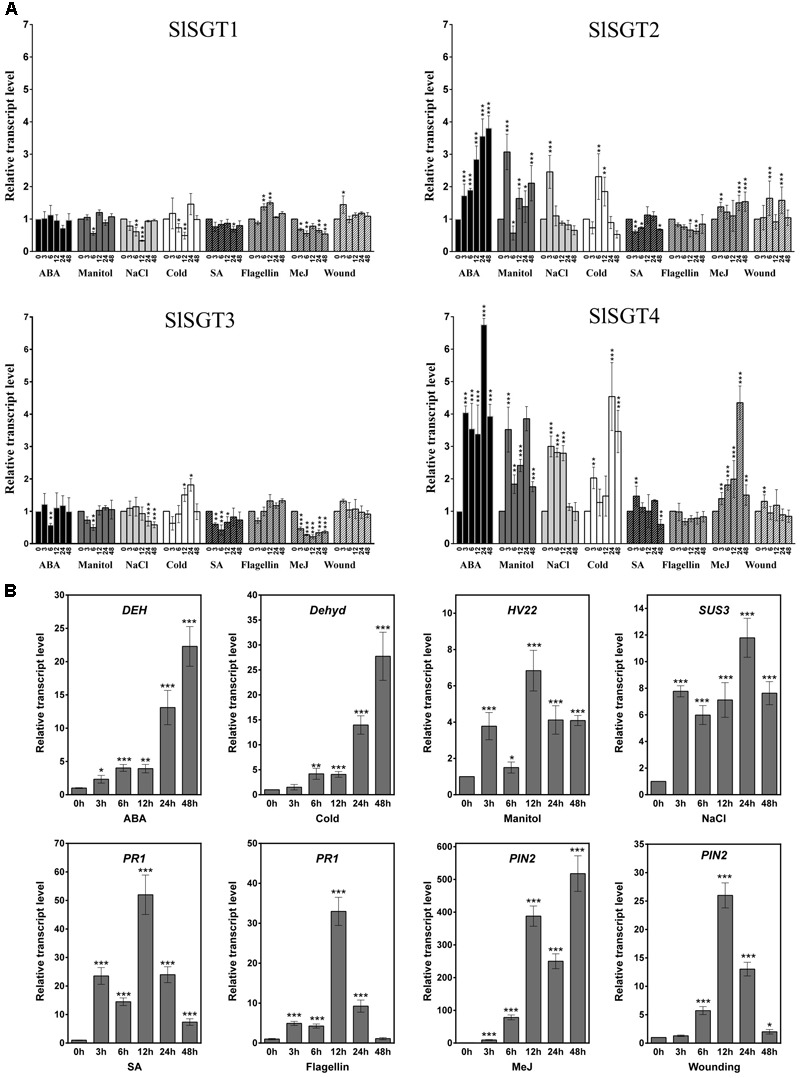
Expression of *SlSGT* genes in tomato seedlings exposed to different stresses. The transcript levels of *SlSGT1, SlSGT2, SlSGT3*, and *SlSGT4* were determined by RT-qPCR using RNA samples from tomato seedlings exposed to different treatments (ABA, mannitol, NaCl, cold, wound, SA, MeJA, and flagellin 22). Samples were collected at the indicated time points (3, 6, 12, 24, and 48 h) from the start of each treatment (0 h) **(A)**. The expression of different marker genes reported as responsive to ABA (*DEH*), cold (*Dehyd*), mannitol (*HVA22*), salt (*SUS3*), SA and flagellin 22 (*PR1*), wound and MeJ (*PIN2)* was analyzed in the same samples **(B)**. Data are expressed as normalized quantity values calculated using three independent housekeeping genes (*PP2Acs* and *EF1a*) (Ballester et al., 2013) and relative to non-treated seedlings at each time point, which is assumed to be one. Values are means ± SD (*n* = 6). Asterisks show the values that are significantly different (^∗^*p* < 0.05, ^∗∗^*p* < 0.01, ^∗∗∗^*p* < 0.001) compared to those at time 0 h.

To gain insight into the possible involvement of tomato SGTs in the plant response to different stresses, we examined the temporal response of the *SlSGT* genes to several stresses, namely osmotic, salt, cold and wound, and stress signals, including flagellin 22 (a pathogen elicitor that activates the plant basal defense response) and the hormones ABA, MeJA, and SA (**Figure 9A**). To this end, the transcript levels of the four *SlSGT* genes were determined by RT-qPCR in RNA samples obtained from 3-week-old tomato seedlings collected before (time point 0 h) and after exposure (time points 3, 6, 12, 24, and 48 h) to the mentioned treatments and compared to those in untreated seedlings collected at the same time points (**Figure 9A**). In order to confirm the activation of the corresponding stress signaling pathways, the transcript levels of different marker genes reported as responsive to the assayed treatments in tomato were measured in the same samples (**Figure 9B**). The significant increases observed in the expression of *DEH* (ABA-inducible dehydrin) (Musser et al., 2012), *Dehyd* (cold-inducible dehydrin) (Weiss and Egea-Cortines, 2009), *HVA22* (osmotic stress-related gene) (Yang et al., 2015), *SUS3* (salt-inducible sucrose synthase) (Rivero et al., 2014), *PR1* (SA- and flagellin-inducible pathogenesis related protein 1) (Gómez-Gómez and Boller, 2002; Molinari et al., 2014) and *PIN2* (wound- and MeJA-inducible proteinase inhibitor 2) (Tian et al., 2014) after exposure of plants to the stress treatments demonstrated that the corresponding signaling pathways had actually been activated (**Figure 9B**). Quantitative RT-qPCR expression analysis of tomato *SlSGTs* showed that the transcript levels of *SlSGT1*, the most actively expressed *SlSGT* gene in adult plant organs (**Figure 5**), remained fairly unaltered in response to the different treatments (**Figure 9A**). A similar expression pattern was observed for *SlSGT3*, though the expression of this gene was repressed upon MeJA treatment (**Figure 9A**). By contrast, the expression of *SlSGT4*, which was hardly detectable in all tested organs of adult plants (**Figure 5**), increased markedly when seedlings were subject to osmotic, salt and cold stress, as well as upon treatment with ABA and MeJA (**Figure 9A**). The strongest induction of *SlSGT4* was observed at time point 24 h in ABA-treated seedlings (about seven-fold higher than basal level). At this time point, the *SlSGT4* transcripts were also significantly up regulated in response to osmotic and cold stress (about four-fold and five-fold, respectively), and to MeJA treatment (about five-fold). It is also remarkable the early induction of this gene by ABA, mannitol, cold and NaCl, which was already observed at 3 h post-treatment (about four-fold for ABA and mannitol, three-fold for NaCl, and two-fold in response to cold). However, while *SlSGT4* expression in response to ABA, mannitol and cold remained higher than in control seedlings throughout the entire time-course analysis, in seedlings treated with NaCl, at time points 24 and 48 h SlSGT4 mRNA levels returned back to basal levels (**Figure 9A**). The expression of *SlSGT4* was not significantly altered in response to wounding, flagellin and SA treatment (**Figure 9A**). The expression profile of *SlSGT2* in response to stress paralleled that of *SlGT4*, although to a lower quantitative degree (**Figure 9A**). Indeed, transcript levels increased significantly in seedlings exposed to ABA, mannitol, cold, and NaCl (**Figure 9A**). A small but significant induction of *SlSGT2* expression was already detected after 3 h of ABA treatment (about two-fold) and increased up to four-fold approximately at time point 48 h. After 3 h, the *SlSGT2* transcripts were also up regulated in response to mannitol (about 3-fold) and salt (about 2.5-fold), and a similar up-regulation was observed after 6 h of seedlings exposure to cold. The expression of this gene was not significantly affected by SA, flagellin, wounding and MeJA (**Figure 9A**). Altogether, these expression results demonstrate that the four members of the *SlSGT* gene family are also differentially regulated in response to biotic and abiotic stress since *SlSGT2* and *SlSGT4* clearly behave as stress-responsive genes whereas *SlSGT1* and *SlSGT3* do not appear to be involved in mediating most of the tomato stress responses.

## Discussion

The results obtained in this study show that the tomato genome harbors a small *SlSGT* gene family of four members that share an almost identical organization with regard to relative intron positions and exon sizes (**Figure 2**) and encode functional SGT isozymes, as demonstrated by the ability of the four recombinant GST-SGT fusion proteins to glycosylate *in vitro* a mixture of FS from UDP-glucose as a sugar moiety donor (**Figure 4**) and the enhanced levels of SG measured in leaves of *N. benthamiana* plants overexpressing the SGT-YFP fusion proteins (**Figure 8E**). The ability of tomato SGTs to glycosylate sterols is fully consistent with the presence in their primary structure of two characteristic structural domains reported to be involved in substrate binding, i.e., the PSBD found in steroid UDP-glucuronosyltransferases and the C-terminal UDP-sugar binding domain (PSPG) characteristic for nucleoside diphosphosugar glycosyltransferases (**Figure 2**). These domains have been also identified in all other functional plant SGTs characterized to date (Supplementary Figure S1) and are included in a conserved core region of 420 amino acid residues that shares 57% overall sequence identity among the four SlSGT proteins. Altogether, the domain organization of tomato SlSGTs fits nicely with that of previously reported SGTs, consisting of a highly conserved central catalytic domain of about 400 amino acid residues preceded by a rather poorly conserved N-terminal extension which appears to be dispensable for *in vitro* catalytic activity (Grille et al., 2010). The ability of the four tomato SGTs to glycosylate the same sterol species *in vitro* (brassicasterol, campesterol, stigmasterol, and β-sitosterol) and *in vivo* (cholesterol, campesterol, stigmasterol, and β-sitosterol) suggests that the tomato enzymes might be functionally redundant, although the possibility that they may exhibit some substrate preferences under physiological conditions cannot be excluded.

The occurrence of small *SGT* gene families seems to be a general feature in plants (Sharma et al., 2007; DeBolt et al., 2009; Chaturvedi et al., 2012; Li et al., 2014), thus raising the question about the possible specialized roles that individual SGT isozymes may play in the metabolism of glycosylated sterols in plants. Our *SlSGT* gene expression studies in different organs of tomato plants, including fruits at different developmental stages, revealed that the *SlSGT* genes, with the apparent exception of *SlSGT4* whose expression was barely detectable, have specialized but still largely overlapping expression patterns (**Figure 5**). This, together with the divergent evolutionary history of SlSGTs depicted by the phylogenetic tree data (**Figure 3**) argues in favor of the hypothesis that tomato SGT isozymes might play overlapping but not completely redundant biological functions. Such specialization could involve different sterol substrate preferences, as demonstrated in the case of Arabidopsis UGT80A2 and UGT80B1 isozymes. UGT80A2 activity seems to account for most of the sitosteryl and stigmasteryl glucoside production in seeds while UGT80B1 preferentially forms brassicasteryl glucosides (DeBolt et al., 2009; Stucky et al., 2015). It is also worth noting that some plant SGTs seem to have broad substrate specificity, as exemplified by the SGTs of *W. somnifera*, a *Solanaceae* species that contains a variety of steroidal aglycones (withanolides), which can be glycosylated along with sterols by members of the WsSGT family (Saema et al., 2016; Singh et al., 2016). WsSGTs have been predicted to interact with a variety of sterols and withanolides but with different substrate preferences. Brassicasterol would be the preferred substrate for *Ws*SGTL1 while *Ws*SGTL3.2 (referred to as *Ws*SGT4 in this study) would prefer withanolide A (Pandey et al., 2015). Thus, the possibility that tomato SGTs might glycosylate other structurally related compounds such as steroidal glycoalkaloids cannot be completely excluded.

The precise subcellular localization of SGTs is one of the most controversial issues regarding steryl glycoside metabolism in eukaryotic organisms including plants. Indeed, a number of studies in various plant species using different experimental approaches have reported multiple subcellular localizations for SGTs, including the cytoplasm (Madina et al., 2007; Grille et al., 2010; Li et al., 2014) and different cell membranes such as PM, ER, Golgi, and tonoplast (Grille et al., 2010; Chaturvedi et al., 2011; Li et al., 2014; Zauber et al., 2014). Our subcellular localization studies using SlSGT-YFP fusions transiently expressed in *N. benthamiana* cells (**Figure 6**) did not allow us to distinguish whether SlSGT-YFP fusions localize in the PM or the cytoplasm, an issue that we further addressed by means of FRAP analysis (**Figures 7, 8A**). FRAP is a technique commonly employed to characterize protein mobility within specific cell compartments that can also be used to determine the subcellular localization of proteins by examining the kinetics of recovery of fluorescence in a previously photobleached area due to diffusion of non-photobleached protein molecules from neighboring areas (Bandyopadhyay et al., 2010; Sorieul et al., 2011; Luu et al., 2012; Jarsch et al., 2014; Misheva et al., 2014; Onrubia et al., 2014; Ramírez-Estrada et al., 2016). FRAP kinetic plots vary depending on whether the protein of interest can freely diffuse through the cytoplasm to replenish the bleached region or has a limited mobility because it is associated or embedded into the more viscous medium of cell membranes, thereby showing a diffusion rate slower than that of soluble proteins. Under our experimental conditions, FRAP curves revealed clear differences in intracellular mobility among the four tomato SGT proteins. The mobility of SlSGT1-YFP, and more notably that of SlSGT3-YFP, was significantly reduced compared to that of cytosolic GFP, although in both cases was clearly higher than that of an intrinsic membrane protein like BRL3 (**Figures 7, 8A** and Supplementary Figure S3), thus suggesting that SlSGT1 and SlSGT3 interact to some extent with the PM. On the contrary, SlSGT2-YFP and SlSGT4-YFP showed fluorescence recovery plots resembling that of GFP (**Figures 7, 8A** and Supplementary Figure S3), which suggested a preferential localization of both proteins in the cytosol. The results of immunoblot analysis of membrane and soluble fractions from *N. benthamiana* cells expressing SlSGT-YFP fusions confirmed the differential distribution of SlSGT-YFP proteins between the cytosol and the PM. SGT3-YFP was almost equally distributed between the two fractions while SlSGT1-YFP, SlSGT2-YFP, and SlSGT4-YFP were primarily detected in the soluble fraction, although it is important to note that the proportion of SlSGT1-YFP in the membrane fraction was greater that that of SlSGT2-YFP and SlSGT4-YFP (**Figure 8B**). We observed a similar concordance between FRAP results and cell fractionation analyses in the case of the Arabidopsis SGTs (**Figures 7, 8B** and Supplementary Figure S3). UGT80A2-YFP, which was primarily detected in the soluble fraction, showed a higher fluorescence recovery rate than UGT80B1-YFP, which was almost equally distributed between the membrane and soluble fractions, similarly to SGT3-YFP (**Figure 8B**). Interestingly, the tomato and Arabidopsis SGT-YFP protein fraction attached to the PM, regardless of the amount, can be dissociated by treatment of the membrane fraction with urea but not with high salt (**Figure 8D**). This, together with the fact that none of these proteins contain any predicted transmembrane domain, strongly support the view that tomato and Arabidopsis SGTs may interact to some extent with the PM, presumably through hydrophobic interactions, but are not deeply immersed in it, thereby behaving as peripheral membrane proteins. This, together with the finding that SGT-YFP proteins expressed in *N. benthamiana* are active in producing SGs (**Figure 8E**), lead us to speculate that tomato and Arabidopsis SGTs are soluble enzymes that may associate with the PM when cells need to enhance the amount of SGs to adjust membrane properties to new environmental and cellular conditions. This hypothesis is compatible with the subcellular localization of their substrates. UDP glucose is a highly polar cytosolic compound whereas sterols are oriented in membranes with the planar ring system embedded in the hydrophobic phase of the lipid bilayer and the free hydroxyl group exposed to the surface of the membrane. Thus, it is conceivable that the catalytic activity of SGTs might require only superficial contact of the enzyme with the membrane, dipping into it to some extent to recognize the hydrophobic sterol backbone (Grille et al., 2010). The possibility that such a transient membrane attachment might involve some lipid-mediated reversible posttranslational modification seems unlikely because no consensus amino acid sequences for myristoylation, palmitoylation, or prenylation can be identified in the SGTs of tomato and Arabidopsis. Only non-consensus prenylation sequences found in Rab proteins (Xie et al., 2016) are detected in the C-terminal end of SlSGT1, SlSGT3, SlSGT4, UGT80A2, and UGT80B1. This, together with the fact that plant SGTs do not posses any obvious lipid binding domain, suggests that recruitment of SGTs to the cell membranes likely involves a different mechanism, as for example an as yet unidentified protein–protein interaction or their own enzyme-substrate recognition, with the catalytic site serving as a lipid binding pocket that recognizes the sterol backbone immersed in the membrane. Regardless of the membrane recruitment mechanism, the differences observed in the intracellular distribution between the cytosol and the PM of tomato SGTs further reinforce the hypothesis that SlSGTs fulfill partially specialized roles in the biosynthesis of SGs. In this respect, it is remarkable that the two *SlSGT* genes that are activated at transcriptional level in response to different environmental stimuli and treatments encode the cytosolic isozymes SlSGT2 and SlSGT4, whereas those that are developmentally regulated code for the membrane-associated enzymes SlSGT1 and SlSGT3.

In their natural environment, plants are generally exposed to several biotic and abiotic stresses. These environmental hazards activate intricate molecular mechanisms that allow plants to perceive the external signals and initiate the optimal defense responses in order to cope with the adverse environmental conditions. These responses are primarily regulated by phytohormones such as ABA, JA, or SA, among others, which may interact synergistically or antagonistically through elaborated networks of signaling pathways that share certain degree of overlap (Fujita et al., 2006). The crosstalk between different stress responses results in lower costs for plant defense, in particular when common genes and compounds are involved in the protective response against multiple stresses (Atkinson and Urwin, 2012), as seems to be the case of sterols and, more specifically, of the FS:SG ratio in the PM (Lynch and Steponkus, 1987; Palta et al., 1993; Moreau et al., 2002; Minami et al., 2009; Grille et al., 2010; Mishra et al., 2013; Li et al., 2014; Pandey et al., 2014; Grosjean et al., 2015; Tarazona et al., 2015; Takahashi et al., 2016). The very high levels of glycosylated sterols found in tomato tissues (Duperon et al., 1984; Whitaker, 1988; Whitaker and Gapper, 2008) along with the important changes observed in their metabolism during chilling and after re-warming (Whitaker, 1991, 1994) and our finding that tomato *SlSGT2* and *SlSGT4* genes are induced when seedlings are exposed to abiotic and biotic stress conditions (**Figure 9**) further support the view that SGTs and, by extension, SGs play a significant role in the metabolic stress response network of tomato.

The rapid induction of *SlSGT4* and *SlSGT2* after cold, mannitol and NaCl treatments (3 h post-treatment) (**Figure 9A**) suggests a role of these genes in the response of tomato plants to abiotic stress. The involvement of SGTs in plant response to abiotic stress is not unprecedented since the expression of the four genes of the *WsSGT* family is also enhanced upon cold treatment, although with a slight delay (Chaturvedi et al., 2012) compared to the tomato genes (**Figure 9A**). The stress-induced expression of specific tomato *SlSGT* genes is also consistent with the response to abiotic stress of mutant plants with altered levels of SGTs. Thus, an Arabidopsis T-DNA insertion mutant defective in the *UGT80B1* gene shows increased sensitivity to cold and heat stress as compared to wild-type plants (Mishra et al., 2015), whereas constitutive expression of *WsSGTL1* in Arabidopsis results in enhanced cold, heat, and salt tolerance (Mishra et al., 2013) and overexpression of the same enzyme in *W. somnifera* and tobacco leads to enhanced tolerance to cold and salt, respectively (Pandey et al., 2014; Saema et al., 2016). Interestingly, the expression of *SlSGT2* and *SlSGT4*, but not that of *SlSGT1* and *SlSGT3*, also increased markedly after 3 h of treatment with ABA (**Figure 9A**), a phytohormone involved in the response to most abiotic stresses (Finkelstein, 2013). The presence of at least one ABRE element in the promoters of *SlSGT4* and *SlSGT2*, which is not found in the promoters of the ABA non-responsive genes *SlSGT1* and *SlSGT3*, (PlantCARE^6^) might explain, at least in part, the differential response of the tomato *SlSGT* genes to abiotic stress and ABA.

The early induction of *SlSGT4* expression after MeJA treatment, which also induced a later and lower activation of *SlSGT2* expression (**Figure 9A**), suggests that MeJA regulates the levels of SG in tomato. A similar response has been reported in *W. somnifera* in which the expression of the different members of the *WsSGT* gene family is also induced by MeJA treatment (Chaturvedi et al., 2012). This suggests that MeJA might be involved in modulating the levels of SG in this species too; although in this case the possibility that up-regulation of *WsSGT* genes in response to MeJA might be related to the glycosylation of withanolides rather than sterols cannot be excluded. In fact, the increased resistance toward *Spodoptera litura* of tobacco plants overexpressing WsSTGL1 (Pandey et al., 2014) and the compromised basal immunity observed upon silencing several members of the WsSGT family (Singh et al., 2016) does not seem attributable to the altered levels of glycosylated sterols but rather to the concomitant change in the levels of rutin (Pandey et al., 2014) and withanolides observed in the corresponding engineered plants (Singh et al., 2016). The high induction of *SlSGT4* expression in response to MeJA (**Figure 9A**) suggests that this gene might be involved in the plant response to necrotrophic pathogens. This is supported by the similar expression profile observed in response to ABA treatment, as this hormone has been identified as a signal required for plant resistance to some necrotrophic pathogens (Adie et al., 2007). In general, treatment with SA has little effect on the expression of the *SlSGT* gene family (**Figure 9A**), suggesting that SA does not directly regulate the levels of SG in tomato. These results, which are different to those observed in *W. somnifera* where the expression of all the *SGT* genes was enhanced by SA (Chaturvedi et al., 2012), suggest that in tomato SGs might be involved in JA-mediated defense rather than in SA-dependent pathways, while both would be involved in *W. somnifera* (Chaturvedi et al., 2012). It remains to be established whether the differential expression pattern of *SGT* genes in response to MeJA and SA in tomato and *W. somnifera* might be related to differences in substrate specificity between the SGTs of these two species, since *W. somnifera* SGTs also glycosylate withanolides in addition to sterols (Saema et al., 2016; Singh et al., 2016). Altogether, these results suggest that, at least in tomato, SGs are involved in plant response to abiotic stress mediated by ABA, although they might also play a role in plant response to biotic stress imposed by necrotrophic pathogens and regulated by JA-dependent pathways.

Overall the results of this study support the hypothesis that the four tomato SGT isoforms perform functions that are overlapping but also specialized, and lay the basis for future work aimed at further exploring the specific contribution of each individual SGT isozyme to SG synthesis and their biological role in tomato.

## Author Contributions

AB, AF, and TA conceived and designed the research; KR-E, NC, JL, and MA carried out the experiments; KR-E, AF, and TA performed data collection, analysis and interpretation, and wrote the manuscript.

## Conflict of Interest Statement

The authors declare that the research was conducted in the absence of any commercial or financial relationships that could be construed as a potential conflict of interest.

## Footnotes

1 www.bio-rad.com

2 http://www.dagexpression.com

3 https://www.arabidopsis.org

4 https://imagej.net

5 https://phytozome.jgi.doe.gov/pz/portal.html

6 http://bioinformatics.psb.ugent.be/webtools/plantcare/html/

## References

[B1] Adie B. A. Pérez-Pérez J. Pérez-Pérez M. M. Godoy M. Sánchez-Serrano J. J. Schmelz E. A. (2007). ABA is an essential signal for plant resistance to pathogens affecting JA biosynthesis and the activation of defenses in *Arabidopsis*. *Plant Cell* 19 1665–1681. 10.1105/tpc.106.048041 17513501 PMC1913739

[B2] Arró M. Manzano D. Ferrer A. (2014). Farnesyl diphosphate synthase assay. *Methods Mol. Biol.* 1153 41–53. 10.1007/978-1-4939-0606-2_4 24777789

[B3] Atkinson N. J. Urwin P. E. (2012). The interaction of plant biotic and abiotic stresses: from genes to the field. *J. Exp. Bot.* 63 3523–3543. 10.1093/jxb/ers100 22467407

[B4] Babiychuk E. Bouvier-Navé P. Compagnon V. Suzuki M. Muranaka T. Van Montagu M. (2008). Allelic mutant series reveal distinct functions for *Arabidopsis* cycloartenol synthase 1 in cell viability and plastid biogenesis. *Proc. Natl. Acad. Sci. U.S.A.* 105 3163–3168. 10.1073/pnas.0712190105 18287026 PMC2268602

[B5] Ballester M. Cordón R. Folch J. M. (2013). DAG expression: high-throughput gene expression analysis of real-time PCR data using standard curves for relative quantification. *PLoS ONE* 8:e80385 10.1371/journal.pone.0080385.s002 PMC3832397 24260380

[B6] Bandyopadhyay A. Kopperud K. Anderson G. (2010). An integrated protein localization and interaction map for Potato yellow dwarf virus, type species of the genus Nucleorhabdovirus. *Virology* 402 61–71. 10.1016/j.virol.2010.03.013 20362316 PMC2873121

[B7] Beck J. G. Mathieu D. Loudet C. Buchoux S. Dufourc E. J. (2007). Plant sterols in “rafts”: a better way to regulate membrane thermal shocks. *FASEB J.* 21 1714–1723. 10.1096/fj.06-7809com 17317727

[B8] Behmer S. T. Olszewski N. Sebastiani J. Palka S. Sparacino G. Sciarrno E. (2013). Plant phloem sterol content: forms, putative functions, and implications for phloem-feeding insects. *Front. Plant Sci.* 4:370 10.3389/fpls.2013.00370 PMC3781331 24069026

[B9] Benveniste P. (2004). Biosynthesis and accumulation of sterols. *Annu. Rev. Plant Biol.* 55 429–457. 10.1146/annurev.arplant.55.031903.141616 15377227

[B10] Blankemeyer J. T. White J. B. Stringer B. K. Friedman M. (1997). Effect of a-tomatine and tomatidine on membrane potential of frog embryos and active transport of ions in frog skin. *Food Chem. Toxicol.* 35 639–646. 10.1016/S0278-6915(97)00038-0 9301646

[B11] Bouvier-Navé P. Berna A. Noiriel A. Compagnon V. Carlsson A. S. Banas A. (2010). Involvement of the phospholipid sterol acyltransferase1 in plant sterol homeostasis and leaf senescence. *Plant Physiol.* 152 107–119. 10.1104/pp.109.145672 19923239 PMC2799350

[B12] Caño-Delgado A. Yin Y. Yu C. Vafeados D. Mora-García S. Cheng J.-C. (2004). BRL1 and BRL3 are novel brassinosteroid receptors that function in vascular differentiation in *Arabidopsis*. *Development* 131 5341–5351. 10.1242/dev.01403 15486337

[B13] Cárdenas P. D. Sonawane P. D. Pollier J. Bossche R. V. Dewangan V. Weithorn E. (2016). GAME9 regulates the biosynthesis of steroidal alkaloids and upstream isoprenoids in the plant mevalonate pathway. *Nat. Commun.* 7 1–16. 10.1038/ncomms10654 PMC4756317 26876023

[B14] Carland F. M. Fujioka S. Takatsuto S. Yoshida S. Nelson T. (2002). The identification of CVP1 reveals a role for sterols in vascular patterning. *Plant Cell* 14 2045–2058. 10.1105/tpc.003939 12215504 PMC150754

[B15] Carruthers A. Melchior D. L. (1986). How bilayer lipids affect membrane-protein activity. *Trends Biochem. Sci.* 11 331–335. 10.1016/0968-0004(86)90292-6

[B16] Chaturvedi P. Mishra M. Akhtar N. Gupta P. Mishra P. Tuli R. (2012). Sterol glycosyltransferases-identification of members of gene family and their role in stress in *Withania somnifera*. *Mol. Biol. Rep.* 39 9755–9764. 10.1007/s11033-012-1841-3 22744427

[B17] Chaturvedi P. Misra P. Tuli R. (2011). Sterol glycosyltransferases-The enzymes that modify sterols. *Appl. Biochem. Biotechnol.* 165 47–68. 10.1007/s11033-012-1841-3 21468635

[B18] Cooke D. T. Burden R. S. (1990). Lipid modulation of plasma membrane-bound ATPases. *Physiol. Plant.* 78 153–159. 10.1111/j.1399-3054.1990.tb08730.x

[B19] Deblaere R. Bytebier B. De Greve H. Deboeck F. Schell J. Van Montagu M. (1985). Efficient octopine Ti plasmid-derived vectors for *Agrobacterium*-mediated gene transfer to plants. *Nucleic Acids Res.* 13 4777–4788. 10.1093/nar/13.13.4777 4022773 PMC321826

[B20] DeBolt S. Scheible W. Schrick K. Auer M. Beisson F. Bischoff V. (2009). Mutations in UDP-glucose:sterol glucosyltransferase in Arabidopsis cause transparent testa phenotype and suberization defects in seeds. *Plant Physiol.* 151 78–87. 10.1104/pp.109.140582 19641030 PMC2735980

[B21] Duperon R. Thiersault M. Duperon P. (1984). High level of glycosilated sterols in species of *Solanum* and sterol changes during the development of the tomato. *Phytochemistry* 23 743–746. 10.1016/S0031-9422(00)85016-5

[B22] Earley K. W. Haag J. R. Pontes O. Opper K. Juehne T. Song K. (2006). Gateway-compatible vectors for plant functional genomics and proteomics. *Plant J.* 45 616–629. 10.1111/j.1365-313X.2005.02617.x 16441352

[B23] Emanuelsson O. Brunak S. von Heijne G. Nielsen H. (2007). Locating proteins in the cell using TargetP, SignalP and related tools. *Nat. Protoc.* 2 953–971. 10.1038/nprot.2007.131 17446895

[B24] Finkelstein R. (2013). Abscisic acid synthesis and response. *Arabidopsis Book* 11:e0166 10.1199/tab.0166 PMC3833200 24273463

[B25] Friedman M. (2002). Tomato glycoalkaloids: role in the plant and in the diet. *J. Agric. Food Chem.* 50 5751–5780. 10.1021/jf020560c 12358437

[B26] Fujita M. Fujita Y. Noutoshi Y. Takahashi F. Narusaka Y. Yamaguchi-Shinozaki K. (2006). Crosstalk between abiotic and biotic stress responses: a current view from the points of convergence in the stress signaling networks. *Cur. Opin. Plant Biol.* 9 436–442. 10.1016/j.pbi.2006.05.014 16759898

[B27] Furt F. König S. Bessoule J.-J. Sargueil F. Zallot R. Stanislas T. (2010). Polyphosphoinositides are enriched in plant membrane rafts and form microdomains in the plasma membrane. *Plant Physiol.* 152 2173–2187. 10.1104/pp.109.149823 20181756 PMC2850013

[B28] Furt F. Simon-Plas F. Mongrand S. (2011). “Lipids of the plant plasma membrane,” in *The Plant Plasma Membranes* eds Murphy A. S. Schulz B. Peer W. (Berlin: Springer) 3–30. 10.1007/978-3-642-13431-9_1

[B29] Gómez-Gómez L. Boller T. (2002). Flagellin perception: a paradigm for innate immunity. *Trends Plant Sci.* 7 251–256. 10.1016/S1360-1385(02)02261-6 12049921

[B30] Goytia E. Fernández-Calvino L. Martínez-García B. López-Abella D. López-Moya J. J. (2006). Production of *Plum pox virus* HC-Pro functionally active for aphid transmission in a transient-expression system. *J. Gen. Virol.* 87 3413–3423. 10.1099/vir.0.82301-0 17030878

[B31] Grandmougin-Ferjani A. Schuler-Muller I. Hartmann M. A. (1997). Sterol modulation of the plasma membrane H+-ATPase activity from corn roots reconstituted into soybean lipids. *Plant Physiol.* 113 163–174. 10.1104/pp.113.1.163 12223599 PMC158127

[B32] Griebel T. Zeier J. (2010). A role for β-sitosterol to stigmasterol conversion in plant-pathogen interactions. *Plant J.* 63 254–268. 10.1111/j.1365-313X.2010.04235.x 20444228

[B33] Grille S. Zaslawski A. Thiele S. Plat J. Warnecke D. (2010). The functions of steryl glycosides come to those who wait: Recent advances in plants, fungi, bacteria and animals. *Prog. Lipid Res.* 49 262–288. 10.1016/j.plipres.2010.02.001 20138912

[B34] Grison M. S. Brocard L. Fouillen L. Nicolas W. Wewer V. Dörmann P. (2015). Specific membrane lipid composition is important for plasmodesmata function in Arabidopsis. *Plant Cell* 27 1228–1250. 10.1105/tpc.114.135731 25818623 PMC4558693

[B35] Grosjean K. Mongrand S. Beney L. Simon-Plas F. Gerbeau-Pissot P. (2015). Differential effect of plant lipids on membrane organization: specificities of phytosphingolipids and phytosterols. *J. Biol. Chem.* 290 5810–5825. 10.1074/jbc.M114.598805 25575593 PMC4342490

[B36] He J.-X. Fujioka S. Li T.-C. Kang S. G. Seto H. Takatsuto S. (2003). Sterols regulate development and gene expression in Arabidopsis. *Plant Physiol.* 131 1258–1269. 10.1104/pp.014605 12644676 PMC166886

[B37] Held M. A. Boulaflous A. Brandizzi F. (2008). Advances in fluorescent protein-based imaging for the analysis of plant endomembranes. *Plant Physiol.* 147 1469–1481. 10.1104/pp.108.120147 18678739 PMC2492624

[B38] Hugly S. McCourt P. Browse J. Patterson G. W. Somerville C. (1990). A chilling sensitive mutant of *Arabidopsis* with altered steryl-ester metabolism. *Plant Physiol.* 93 1053–1062. 10.1104/pp.93.3.1053 16667557 PMC1062630

[B39] Iijima Y. Watanabe B. Sasaki R. Takenaka M. Ono H. Sakurai N. (2013). Steroidal glycoalkaloid profiling and structures of glycoalkaloids in wild tomato fruit. *Phytochemistry* 95 145–157. 10.1016/j.phytochem.2013.07.016 23941899

[B40] Ischebeck T. (2016). Lipids in pollen - They are different. *Biochim. Biophys. Acta* 1861 1315–1328. 10.1016/j.bbalip.2016.03.023 27033152

[B41] Ishikawa-Ankerhold H. C. Ankerhold R. Drummen G. P. C. (2012). Advanced fluorescence microscopy techniques-FRAP, FLIP, FLAP, FRET and FLIM. *Molecules* 17 4047–4132. 10.3390/molecules17044047 22469598 PMC6268795

[B42] Itkin M. Rogachev I. Alkan N. Rosenberg T. Malitsky S. Masini L. (2012). GLYCOALKALOID METABOLISM1 is required for steroidal alkaloid glycosylation and prevention of phytotoxicity in tomato. *Plant Cell* 23 4507–4525. 10.1105/tpc.111.088732 PMC3269880 22180624

[B43] Jang J. Fujioka S. Tasaka M. Seto H. Takatsuto S. Ishii A. (2000). A critical role of sterols in embryonic patterning and meristem programming revealed by the fackel mutants of *Arabidopsis thaliana*. *Genes Dev.* 14 k1485–1497. 10.1101/gad.14.12.1485 PMC316687 10859167

[B44] Jarsch I. K. Konrad S. S. Stratil T. F. Urbanus S. L. Szymanski W. Braun P. (2014). Plasma membranes are subcompartmentalized into a plethora of coexisting and diverse microdomains in *Arabidopsis* and *Nicotiana benthamiana*. *Plant Cell* 26 1698–1711. 10.1105/tpc.114.124446 24714763 PMC4036580

[B45] Keukens E. A. de Vrije T. van den Boom C. de Waard P. Plasman H. H. Thiel F. (1995). Molecular basis of glycoalkaloid induced membrane disruption. *Biochim. Biophys. Acta* 1240 216–228. 10.1016/0005-2736(95)00186-7 8541293

[B46] Kim H. B. Lee H. Oh C. J. Lee H.-Y. Eum H. L. Kim H.-S. (2010). Postembryonic seedling lethality in the sterol-deficient Arabidopsis cyp51A2 mutant is partially mediated by the composite action of ethylene and reactive oxygen species. *Plant Physiol.* 152 192–205. 10.1104/pp.109.149088 19915013 PMC2799356

[B47] Kopischke M. Westphal L. Schneeberger K. Clark R. Ossowski S. Wewer V. (2013). Impaired sterol ester synthesis alters the response of *Arabidopsis thaliana* to *Phytophthora infestans*. *Plant J.* 73 456–468. 10.1111/tpj.12046 23072470

[B48] Krogh A. Larsson B. Von Heijne G. Sonnhammer E. L. (2001). Predicting transmembrane protein topology with a hidden Markov model: application to complete genomes. *J. Mol. Biol.* 305 567–580. 10.1006/jmbi.2000.4315 11152613

[B49] Kumar M. S. S. Ali K. Dahuja A. Tyagi A. (2015). Role of phytosterols in drought stress tolerance in rice. *Plant Physiol. Biochem.* 96 83–89. 10.1016/j.plaphy.2015.07.014 26233709

[B50] Kumar S. Stecher G. Tamura K. (2016). Molecular evolutionary genetic analysis version 7.0 for bigger datasets. *Mol. Biol. Evol.* 33 1870–1874. 10.1093/molbev/msv054 27004904 PMC8210823

[B51] Laemmli U. K. (1970). Cleavage of structural proteins during the assembly of the head of bacteriophage T4. *Nature* 227 680–685. 10.1038/227680a0 5432063

[B52] Laloi M. Perret A. M. Chatre L. Melser S. Cantrel C. Vaultier M. N. (2007). Insights into the role of specific lipids in the formation and delivery of lipid microdomains to the plasma membrane of plant cells. *Plant Physiol.* 143 461–472. 10.1104/pp.106.091496 17114270 PMC1761958

[B53] Lefebvre B. Furt F. Hartmann M. A. Michaelson L. V. Carde J. P. Sargueil-Boiron F. (2007). Characterization of lipid rafts from Medicago truncatula root plasma membranes: a proteomic study reveals the presence of a raft-associated redox system. *Plant Physiol.* 144 402–418. 10.1104/pp.106.094102 17337521 PMC1913791

[B54] Li R. Sun R. Hicks G. R. Raikhel N. V. (2015). Arabidopsis ribosomal proteins control vacuole trafficking and developmental programs through the regulation of lipid metabolism. *Proc. Natl. Acad. Sci. U.S.A.* 112 E89–E98. 10.1073/pnas.1422656112 25535344 PMC4291620

[B55] Li X. Xia T. Huang J. Guo K. Liu X. Chen T. (2014). Distinct biochemical activities and heat shock responses of two UDP-glucose sterol glucosyltransferases in cotton. *Plant Sci.* 219-220 1–8. 10.1016/j.plantsci.2013.12.013 24576758

[B56] Livak K. J. Schmittgen T. D. (2001). Analysis of relative gene expression data using real-time quantitative PCR and the 2(-Delta Delta C(T)) method. *Methods* 25 402–408. 10.1006/meth.2001.1262 11846609

[B57] Luu D. T. Martinière A. Sorieul M. Runions J. Maurel C. (2012). Fluorescence recovery after photobleaching reveals high cycling dynamics of plasma membrane aquaporins in Arabidopsis roots under salt stress. *Plant J.* 69 894–905. 10.1111/j.1365-313X.2011.04841.x 22050464

[B58] Lynch D. V. Criss A. K. Lehoczky J. L. Bui V. T. (1997). Ceramide glucosylation in bean hypocotyl microsomes: evidence that steryl glucoside serves as glucose donor. *Arch. Biochem. Biophys.* 340 311–316. 10.1006/abbi.1997.9928 9143336

[B59] Lynch D. V. Steponkus P. L. (1987). Plasma membrane lipid alterations associated with cold acclimation of winter rye seedlings (*Secale cereale* L. cv Puma). *Plant Physiol.* 83 761–767. 10.1104/pp.83 16665335 PMC1056446

[B60] Madina B. R. Sharma L. K. Chaturvedi P. Sangwan R. S. Tuli R. (2007). Purification and physico-kinetic characterization of 3beta-hydroxy specific sterol glucosyltransferase from *Withania somnifera* (L) and its stress response. *Biochim. Biophys. Acta* 1774 392–402. 10.1016/j.bbapap.2006.12.009 17293176

[B61] Malinsky J. Opekarová M. Grossmann G. Tanner W. (2013). Membrane microdomains, rafts, and detergent-resistant membranes in plants and fungi. *Annu. Rev. Plant Biol.* 64 501–529. 10.1146/annurev-arplant-050312-120103 23638827

[B62] Manzano D. Andrade P. Caudepón D. Altabella T. Arró M. Ferrer A. (2016). Suppressing Farnesyl diphosphate synthase alters chloroplast development and triggers sterol-dependent induction of jasmonate- and Fe-related responses. *Plant Physiol.* 172 93–117. 10.1104/pp.16.00431 27382138 PMC5074618

[B63] Men S. Boutté Y. Ikeda Y. Li X. Palme K. Stierhof Y.-D. (2008). Sterol-dependent endocytosis mediates post-cytokinetic acquisition of PIN2 auxin efflux carrier polarity. *Nat. Cell Biol.* 10 237–244. 10.1038/ncb1686 18223643

[B64] Minami A. Fujiwara M. Furuto A. Fukao Y. Yamashita T. Kamo M. (2009). Alterations in detergent-resistant plasma membrane microdomains in *Arabidopsis thaliana* during cold acclimation. *Plant Cell Physiol.* 50 341–359. 10.1093/pcp/pcn202 19106119

[B65] Misheva M. Kaur G. Ngoei K. R. Yeap Y. Y. Ng I. H. Wagstaff K. M. (2014). Intracellular mobility and nuclear trafficking of the stress-activated kinase JNK1 are impeded by hyperosmotic stress. *Biochim. Biophys. Acta* 1843 253–264. 10.1016/j.bbamcr.2013.10.017 24184208

[B66] Mishra M. K. Chaturvedi P. Singh R. Singh G. Sharma L. K. Pandey V. (2013). Overexpression of WsSGTL1 gene of *Withania somnifera* enhances salt tolerance, heat tolerance and cold acclimation ability in transgenic *Arabidopsis* plants. *PLoS ONE* 8:e63064 10.1371/journal.pone.0063064.s015 PMC3639950 23646175

[B67] Mishra M. K. Singh G. Tiwari S. Singh R. Kumari N. Misra P. (2015). Characterization of *Arabidopsis* sterol glycosyltransferase TTG15/UGT80B1 role during freeze and heat stress. *Plant Signal. Behav.* 10:e1075682 10.1080/15592324.2015.1075682 PMC4854349 26382564

[B68] Molinari S. Fanelli E. Leonetti P. (2014). Expression of tomato salicylic acid (SA)-responsive pathogenesis-related genes in Mi-1-mediated and SA-induced resistance to root-knot nematodes. *Mol. Plant Path.* 15 255–264. 10.1111/mpp.12085 24118790 PMC6638815

[B69] Moreau R. A. Whitaker B. D. Hicks K. B. (2002). Phytosterols, phytostanols, and their conjugates in foods: structural diversity, quantitative analysis, and health-promoting uses Prog. *Lipid Res.* 41 457–500. 10.1016/S0163-7827(02)00006-1 12169300

[B70] Musser R. O. Hum-Musser S. M. Lee H. K. DesRochers B. L. Williams S. A. Vogel H. (2012). Caterpillar labial saliva alters tomato plant gene expression. *J. Chem. Ecol.* 38 1387–1401. 10.1007/s10886-012-0198-3 23065106

[B71] Nyström L. Schär A. Lampi A.-M. (2012). Steryl glycosides and acylated steryl glycosides in plant foods reflect unique sterol patterns. *Eur. J. Lipid Sci. Technol.* 114 656–669. 10.1002/ejlt.201200033

[B72] Onrubia M. Pollier J. Vanden Bossche R. Goethals M. Gevaert K. Moyano E. (2014). Taximin, a conserved plant-specific peptide is involved in the modulation of plant-specialized metabolism. *Plant Biotechnol. J.* 12 971–983. 10.1111/pbi.12205 24852175

[B73] Palta J. P. Whitaker B. D. Weiss L. S. (1993). Plasma membrane lipids associated with genetic variability in freezing tolerance and cold acclimation of *Solanum* species. *Plant Physiol.* 103 793–803. 10.1104/pp.103.3.793 12231980 PMC159049

[B74] Pandey V. Dhar Y. V. Gupta P. Bag S. K. Atri N. Asif M. H. (2015). Comparative interactions of withanolides and sterols with two members of sterolglycosyltransferases from *Withania somnifera*. *BMC Bioinformatics* 16:120 10.1186/s12859-015-0563-7 PMC4407318 25888493

[B75] Pandey V. Niranjan A. Atri N. Chandrashekhar K. Mishra M. K. Trivedi P. K. (2014). WsSGTL1 gene from *Withania somnifera*, modulates glycosylation profile, antioxidant system and confers biotic and salt stress tolerance in transgenic tobacco. *Planta* 239 1217–1231. 10.1007/s00425-014-2046-x 24610300

[B76] Peng L. Kawagoe Y. Hogan P. Delmer D. (2002). Sitosterol-β-glucoside as primer for cellulose synthesis in plants. *Science* 295 147–150. 10.1126/science.1064281 11778054

[B77] Posé D. Castanedo I. Borsani O. Nieto B. Rosado A. Taconnat L. (2009). Identification of the *Arabidopsis* dry2/sqe1-5 mutant reveals a central role for sterols in drought tolerance and regulation of reactive oxygen species. *Plant J.* 59 63–76. 10.1111/j.1365-313X.2009.03849.x 19309460

[B78] Qian P. Han B. Forestier E. Hu Z. Gao N. Lu W. (2013). Sterols are required for cell-fate commitment and maintenance of the stomatal lineage in Arabidopsis. *Plant J.* 74 1029–1044. 10.1111/tpj.12190 23551583

[B79] Ramírez-Estrada K. Altabella T. Onrubia M. Moyano E. Notredame C. Osuna L. (2016). Transcript profiling of jasmonate-elicited *Taxus* cells reveals a β-phenylalanine-CoA ligase. *Plant Biotechnol. J.* 14 85–96. 10.1111/pbi.12359 25899320 PMC11389183

[B80] Reits E. A. Neefjes J. J. (2001). From fixed to FRAP: measuring protein mobility and activity in living cells. *Nat. Cell Biol.* 3 E145–E147. 10.1038/35078615 11389456

[B81] Rivero R. M. Mestre T. C. Mittler R. O. N. Rubio F. Garcia-Sanchez F. Martinez V. (2014). The combined effect of salinity and heat reveals a specific physiological, biochemical and molecular response in tomato plants. *Plant Cell Environ.* 37 1059–1073. 10.1111/pce.12199 24028172

[B82] Roche Y. Gerbeau-Pissot P. Buhot B. Thomas D. Bonneau L. Gresti J. (2008). Depletion of phytosterols from the plant plasma membrane provides evidence for disruption of lipid rafts. *FASEB J.* 22 3980–3991. 10.1096/fj.08-111070 18676403

[B83] Saema S. Rahman L. U. Singh R. Niranjan A. Ahmad I. Z. Misra P. (2016). Ectopic overexpression of WsSGTL1, a sterol glucosyltransferase gene in *Withania somnifera*, promotes growth, enhances glycowithanolide and provides tolerance to abiotic and biotic stresses. *Plant Cell Rep.* 35 195–211. 10.1007/s00299-015-1879-5 26518426

[B84] Schaller H. (2003). The role of sterols in plant growth and development. *Prog. Lipid Res.* 42 163–175. 10.1016/S0163-7827(02)00047-4 12689617

[B85] Schaller H. (2004). New aspects of sterol biosynthesis in growth and development of higher plants. *Plant Physiol. Biochem.* 42 465–476. 10.1016/j.plaphy.2004.05.012 15246059

[B86] Schrick K. Debolt S. Bulone V. (2012). Deciphering the molecular functions of sterols in cellulose biosynthesis. *Front. Plant Sci.* 3:84 10.3389/fpls.2012.00084 PMC3355633 22639668

[B87] Senthil-Kumar M. Wang K. Mysore K. S. (2013). AtCYP710A1 gene-mediated stigmasterol production plays a role in imparting temperature stress tolerance in *Arabidopsis thaliana*. *Plant Signal. Behav.* 8:e23142 10.4161/psb.23142 PMC3657011 23299431

[B88] Sharma L. K. Madina B. R. Chaturvedi P. Sangwan R. S. Tuli R. (2007). Molecular cloning and characterization of one member of 3beta-hydroxy sterol glucosyltransferase gene family in *Withania somnifera*. *Arch. Biochem. Biophys.* 460 48–55. 10.1016/j.abb.2007.01.024 17324374

[B89] Singh G. Tiwari M. Singh S. P. Singh S. Trivedi P. K. Misra P. (2016). Silencing of sterol glycosyltransferases modulates the withanolide biosynthesis and leads to compromised basal immunity of *Withania somnifera*. *Sci. Rep.* 6:25562 10.1038/srep25562 PMC4857139 27146059

[B90] Sonawane P. D. Pollier J. Panda S. Szymanski J. Massalha H. Yona M. (2016). Plant cholesterol biosynthetic pathway overlaps with phytosterol metabolism. *Nat. Plants* 3:16205 10.1038/nplants.2016.205 28005066

[B91] Sorieul M. Santoni V. Maurel C. Luu D. T. (2011). Mechanisms and effects of retention of over-expressed aquaporin AtPIP2;1 in the endoplasmic reticulum. *Traffic* 12 473–482. 10.1111/j.1600-0854.2010.01154.x 21182578

[B92] Souter M. Topping J. Pullen M. Friml J. Palme K. Hackett R. (2002). hydra mutants of Arabidopsis are defective in sterol profiles and auxin and ethylene signaling. *Plant Cell* 14 1017–1031. 10.1105/tpc.001248 12034894 PMC150604

[B93] Steel C. C. Drysdale R. B. (1988). Electrolyte leakage from plant and fungal tissues and disruption of liposome membranes by α-tomatine. *Phytochemistry* 27 1025–1030. 10.1016/0031-9422(88)80266-8

[B94] Stucky D. F. Arpin J. C. Schrick K. (2015). Functional diversification of two UGT80 enzymes required for steryl glucoside synthesis in *Arabidopsis*. *J. Exp. Bot.* 66 189–201. 10.1093/jxb/eru410 25316063 PMC4265157

[B95] Takahashi D. Imai H. Kawamura Y. Uemura M. (2016). Lipid profiles of detergent resistant fractions of the plasma membrane in oat and rye in association with cold acclimation and freezing tolerance. *Cryobiology* 72 123–134. 10.1016/j.cryobiol.2016.02.003 26904981 PMC5085965

[B96] Tanner W. Opekarová M. Malinsky J. (2011). In plant and animal cells, detergent-resistant membranes do not define functional membrane rafts. *Plant Cell* 23 1191–1193. 10.1105/tpc.111.086249 21531862 PMC3101544

[B97] Tarazona P. Feussner K. Feussner I. (2015). An enhanced plant lipidomics method based on multiplexed liquid chromatography–mass spectrometry reveals additional insights into cold- and drought-induced membrane remodeling. *Plant J.* 84 621–633. 10.1111/tpj.13013 26340975

[B98] Tian D. Peiffer M. De Moraes C. M. Felton G. W. (2014). Roles of ethylene and jasmonic acid in systemic induced defense in tomato (*Solanum lycopersicum*) against *Helicoverpa zea*. *Planta* 239 577–589. 10.1007/s00425-013-1997-7 24271004

[B99] Tiwari P. Sangwan R. S. Asha Mishra B. N. Sabir F. Sangwan N. S. (2014). Molecular cloning and biochemical characterization of a recombinant sterol 3-O-glucosyltransferase from *Gymnema sylvestre* R.Br. catalyzing biosynthesis of steryl glucosides. *BioMed. Res. Int.* 2014:934351 10.1155/2014/934351 PMC4163426 25250339

[B100] Uemura M. Steponkus P. L. (1994). A contrast of the plasma membrane lipid composition of oat and rye leaves in relation to freezing tolerance. *Plant Physiol.* 104 479–496. 10.1104/pp.104.2.479 12232097 PMC159222

[B101] Urbany C. Benke A. Marsian J. Huettel B. Reinhardt R. Stich B. (2013). Ups and downs of a transcriptional landscape shape iron deficiency associated chlorosis of the maize inbreds B73 and Mo17. *BMC Plant Biol.* 13:213 10.1186/1471-2229-13-213 PMC3881016 24330725

[B102] Wagatsuma T. Khan M. S. Watanabe T. Maejima E. Sekimoto H. Yokota T. (2015). Higher sterol content regulated by CYP51 with concomitant lower phospholipid content in membranes is a common strategy for aluminium tolerance in several plant species. *J. Exp. Bot.* 66 907–918. 10.1093/jxb/eru455 25416794 PMC4321553

[B103] Wang H. Nagegowda D. A. Rawat R. Bouvier-Navé P. Guo D. Bach T. J. (2012). Overexpression of *Brassica juncea* wild-type and mutant HMG-CoA synthase 1 in Arabidopsis up-regulates genes in sterol biosynthesis and enhances sterol production and stress tolerance. *Plant Biotechnol. J.* 10 31–42. 10.1111/j.1467-7652.2011.00631.x 21645203

[B104] Wang K. Senthil-Kumar M. Ryu C.-M. Kang L. Mysore K. S. (2012). Phytosterols play a key role in plant innate immunity against bacterial pathogens by regulating nutrient efflux into the apoplast. *Plant Physiol.* 158 1789–1802. 10.1104/pp.111.189217 22298683 PMC3320186

[B105] Warnecke D. C. Baltrusch M. Buck F. Wolter F. P. Heinz E. (1997). UDP-glucose:sterol glucosyltransferase: cloning and functional expression in *Escherichia coli*. *Plant Mol. Biol.* 35 597–603. 10.1023/A:1005806119807 9349281

[B106] Weiss J. Egea-Cortines M. (2009). Transcriptomic analysis of cold response in tomato fruits identifies dehydrin as a marker of cold stress. *J. Appl. Genet.* 50 311–319. 10.1007/BF03195689 19875881

[B107] Whitaker B. D. (1988). Changes in the steryl lipid content and composition of tomato fruit during ripening. *Phytochemistry* 27 3411–3416. 10.1016/0031-9422(88)80740-4

[B108] Whitaker B. D. (1991). Changes in lipids of tomato fruit stored at chilling and non-chilling temperatures. *Phytochemistry* 30 757–761. 10.1016/0031-9422(91)85247-W

[B109] Whitaker B. D. (1994). Lipid changes in mature-green tomatoes during ripening, during chilling, and after rewarming subsequent to chilling. *J. Am. Soc. Hort. Sci.* 119 994–999. 10.1111/j.1399-3054.1991.tb02943.x

[B110] Whitaker B. D. Gapper N. E. (2008). Ripening-specific stigmasterol increase in tomato fruit is associated with increased sterol C-22 desaturase (CYP710A11) gene expression. *J. Agric. Food Chem.* 56 3828–3835. 10.1021/jf7037983 18461960

[B111] Wojciechowski Z. A. (1991). “Biochemistry of phytosterol conjugates,” in *Physiology and Biochemistry of Sterols* eds Patterson G. W. Nes W. D. (Champaign IL: American Oil Chemists’ Society) 361–394. 10.1201/9781439821831.ch14

[B112] Wu Y.-X. Masison D. C. Eisenberg E. Greene L. E. (2006). Application of photobleaching for measuring diffusion of prion proteins in cytosol of yeast cells. *Methods* 39 43–49. 10.1016/j.ymeth.2006.04.004 16793282 PMC3169331

[B113] Xie Y. Zheng Y. Li H. Luo X. He Z. Cao S. (2016). GPS-Lipid: a robust tool for the prediction of multiple lipid modification sites. *Sci. Rep.* 6:28249 10.1038/srep28249 PMC4910163 27306108

[B114] Yang Y. Tang N. Xian Z. Li Z. (2015). Two SnRK2 protein kinases genes play a negative regulatory role in the osmotic stress response in tomato. *Plant Cell Tiss. Organ Cult.* 122 421–434. 10.1007/s11240-015-0779-2

[B115] Yokota T. (1997). The structure, biosynthesis and function of brassinosteroids. *Trends Plant Sci.* 2 137–143. 10.1016/S1360-1385(97)01017-0

[B116] Zauber H. Burgos A. Garapati P. Schulze W. X. (2014). Plasma membrane lipid–protein interactions affect signaling processes in sterol-biosynthesis mutants in *Arabidopsis thaliana*. *Front. Plant Sci.* 5:78 10.3389/fpls.2014.00078014.00078 PMC3957024 24672530

