# Supplementary material for: Tomato UDP-Glucose Sterol Glycosyltransferases: A Family of Developmental and Stress Regulated Genes that Encode Cytosolic and Membrane-Associated Forms of the Enzyme
Source: Front Plant Sci. 2017 Jun 9;8:984. doi: 10.3389/fpls.2017.00984 (PMC5465953; doi:10.3389/fpls.2017.00984)
Supplement: Supplementary file 1 [file Table_1.PDF]

Supplemental Table 1

Supplemental Table 1: SlSGT gene specific primers used for cloning and heterologous expression of SlSGT coding sequences fused to GST and YFP. *Bam*HI and *Sma*I restriction sites are underlined and the ATG start codons are shown in bold.

**Primer name**	**Primer sequence**	**Application**
SlSGT1-fw	CACCTT**ATG**GATCAAACTTCGCCG	Amplification of SlSGT open reading frames for cloning into pENTR/D-TOPO vector.
SlSGT1-rev	CACTCAGGTCGAGGAACACCC	
SlSGT2-fw	CACCAAGTATCA**ATG**TCGAAAGAG	
SlSGT2-rev	ATTTCACACATTGAGATCTAGCA	
SlSGT3-fw	CACCGAA**ATG**GATAGTAGTGGGCTTAATG	
SlSGT3-rev	TCAGAGCCAAGAACCACAAGGCAG	
SlSGT4-fw	CACC**ATG**GACGATTCGTTGGAAAAG	
SlSGT4-rev	GTACCTAGGTTACACATCCAGCGAC	
SlSGT1pgx-fw	**ATG**GATCAAACTTCGCCG	Amplification of SlSGT open reading frames for cloning into pGEX-NotI expression vector.
SlSGT1pgx-rev	CGCGGATCCTCAGGTCGAGGAACACCC	
SlSGT2pgx-fw	**ATG**TCGAAAGAGAAGACTGTTGTAG	
SlSGT2pgx-rev	CGCGGATCCCTAGCAAAAACCTAAGCATCTTC	
SlSGT3pgx-fw	**ATG**GATAGTAGTGGGCTTAATGG	
SlSGT3pgx-rev	TCCCCCGGGTCAGAGCCAAGAACCACAAG	
SlSGT4pgx-fw	**ATG**GACGATTCGTTGGAAAAG	
SlSGT4pgx-rev	CGCGGATCCTTAGGAGTGTCCGATACAGCGTC	
C-SGT1-fw	CACC**ATG**GATCAAACTTCGCCGG	Amplification of SlSGT coding sequences to generate C-terminal fusions with YFP.
C-SGT1-rev	GGTCGAGGAACACCCAAAG	
C-SGT2-fw	CACC**ATG**TCGAAAGAGAAGACTGTTG	
C-SGT2-rev	GCAAAAACCTAAGCATCTTC	
C-SGT3-fw	CACC**ATG**GATAGTAGTGGGCTTAATG	
C-SGT3-rev	GAGCCAAGAACCACAAGGC	
C-SGT4-fw	CACC**ATG**GACGATTCGTTGGAAAAG	
C-SGT4-rev	GGAGTGTCCGATACAGCGTC	
