# Supplementary material for: Tomato UDP-Glucose Sterol Glycosyltransferases: A Family of Developmental and Stress Regulated Genes that Encode Cytosolic and Membrane-Associated Forms of the Enzyme
Source: Front Plant Sci. 2017 Jun 9;8:984. doi: 10.3389/fpls.2017.00984 (PMC5465953; doi:10.3389/fpls.2017.00984)
Supplement: Supplementary file 2 [file Table_2.PDF]

Supplemental Table 2

Supplemental Table 2. Primer sequences used in qPCR and high-throughput quantitative expression analyses of tomato *SlSGT* genes.

Gene	Forward primer	Reverse primer
SGT1	AACGAGCCTCCACGATTGCCTAAA	CTTTCTGGCATAAGAGCACCGCTA
SGT2	GATGAAACACCTGCACCACGAAGT	GGTATCCAACTATTTAGGGTAGCAATG
SGT3	ATCTGTTGCCTGCCTTGT	GATGTGATGAACCAATACAACCAA
SGT4	TGCGTCTTCCTCCACCT	GGGTATCCTTATCTTTCTCAGAATCTA
HVA22	GATATTTGTGGCATGGCTAGTT	TTGGATTTGGCTTTAGGAGAC
LeSUS3	TTGGATTTTGAGCCCTTCAC	AGAGAGGTGCCTGTTGAGGA
Dehyd	TCACAAGGAAGAATCGAAAGC	GACACCACCTCCGTCTTGTTATG
PIN2	CAAGATGTCCCCGTTCACA	CACTCTCTCCTTCACATACAAACT
PR1b1	GTAGGCAACTGGATCGGACAAC	TCATATTAGCAACATCAAAAGGGAA
DEH	GCAAGACTGATGAATATGGAAACC	TACCAGTACCCATGCCTTGAG
Actin	CCTTCCACATGCCATTCTCC	CCACGCTCGGTCAGGATCT
EIF-1a	GAACTGTCCCAGTTGGTCGT	GTCAAACCAGTAGGGCCAAA
UBI	TCGTAAGGAGTGCCCTAATGCTGA	CAATCGCCTCCAGCCTTGTTGTAA
GAPDH	ACCACAAATTGCCTTGCTCCCTTG	ATCAACGGTCTTCTGAGTGGCTGT
TIP41	ATGGAGTTTTTGAGTCTTCTGC	GCTGCGTTTCTGGCTTAGG
PP2Acs	CGATGTGTGATCTCCTATGGTC	AAGCTGATGGGCTCTAGAAATC
Expressed	GCTAAGAACGCTGGACCTAATG	TGGGTGTGCCTTTCTGAATG
EF1	ATTGGAAATGGATATGCTCCA	TCCTTACCTGAACGCCTGTCA
CAC	CACCCTCCGTTGTGATGTAACTGG	ATTGGTGGAAAGTAACATCATCG
