# Supplementary material for: Tomato UDP-Glucose Sterol Glycosyltransferases: A Family of Developmental and Stress Regulated Genes that Encode Cytosolic and Membrane-Associated Forms of the Enzyme
Source: Front Plant Sci. 2017 Jun 9;8:984. doi: 10.3389/fpls.2017.00984 (PMC5465953; doi:10.3389/fpls.2017.00984)
Supplement: Supplementary file 3 [file Table_3.PDF]

Supplemental Table 3

Supplemental Table 3. The upper table shows the overall identity (blue background) and similarity (gray background) percentages shared between de four tomato SGT proteins. The lower table shows the percentages of overall identity between tomato SGTs and functional SGTs from different plant species including *Arabidopsis thaliana* UGT80A2 (Z83833) and UGT80B1 (BT005834), *Withania somnifera* SGT1, (DQ356887), SGT3.1 (EU342379), SGT3.2 (EU342374) and SGT3.3 (EU342375), *Gossypium hirsutum* SGT1 (KJ572778) and SGT2 (KJ572779) and *Avena sativa* SGT (Z83832). Percentages were calculated from pairwise alignment of the corresponding amino acid sequences using the BLASTP algorithm at the NCBI.

	SlSGT1	SlSGT2	SlSGT3	SlSGT4
SlSGT1		80	59	65
SlSGT2	85		59	74
SlSGT3	73	75		50
SlSGT4	77	85	64	

	SlSGT1	SlSGT2	SlSGT3	SlSGT4
UGT80A2	71	72	56	71
UGT80B1	58	56	69	51
WsSGT1	58	58	85	57
WsSGT3.1	66	73	57	87
WsSGT3.2	64	70	55	84
WsSGT3.3	77	79	61	91
GhSGT1	72	74	55	68
GhSGT2	55	61	73	56
AsSGT	68	71	57	69
