# Supplementary material for: Tomato UDP-Glucose Sterol Glycosyltransferases: A Family of Developmental and Stress Regulated Genes that Encode Cytosolic and Membrane-Associated Forms of the Enzyme
Source: Front Plant Sci. 2017 Jun 9;8:984. doi: 10.3389/fpls.2017.00984 (PMC5465953; doi:10.3389/fpls.2017.00984)
Supplement: Supplementary file 4 [file Image_1.PDF]

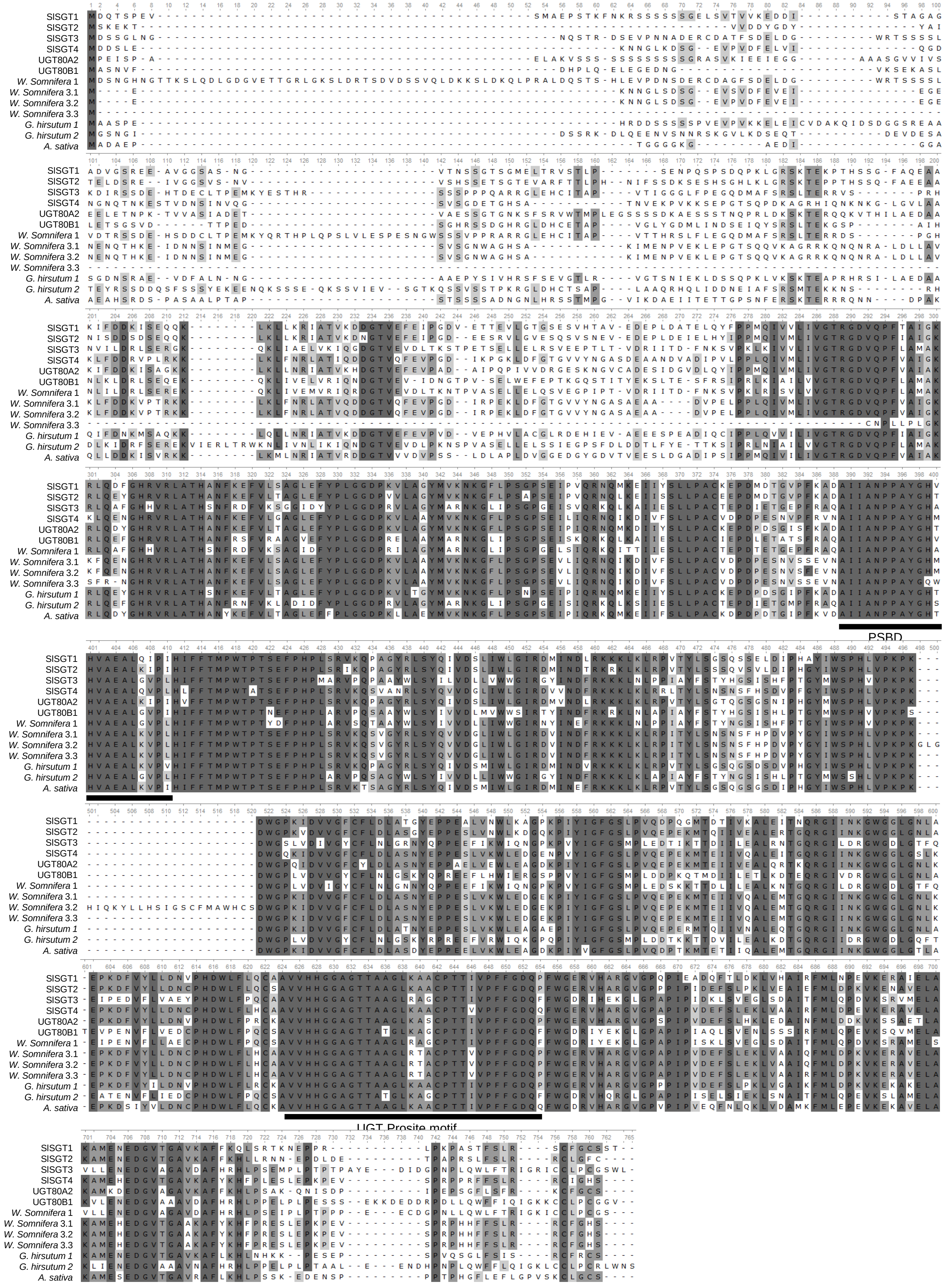
SlSGT1
SlSGT2
SlSGT3
SlSGT4
UGT80A2
UGT80B1
W. Somnifera 1
W. Somnifera 3.1
W. Somnifera 3.2
W. Somnifera 3.3
G. hirsutum 1
G. hirsutum 2
A. sativa
PSBD
UGT Prosite motif

Figure S1. Multiple sequence alignment of *Solanum lycopersicum* cv. Micro-Tom SGT protein sequences (SlSGT1 to 4) and SGTs from different plant species including *A. thaliana* UGT80A2 (Z83833) and UGT80B1 (BT005834), *W. somnifera* SGT1, (DQ356887), SGT3.1 (EU342379), SGT3.2 (EU342374) and SGT3.3 (EU342375), *G. hirsutum* SGT1 (KJ572778) and SGT2 (KJ572779) and *A. sativa* SGT (Z83832). The predicted sterol binding domain (PSBD) and the UGT prosite motif present in the aligned SGT sequences are underlined. Black letters on dark gray background indicate completely conserved residues and less conserved residues are indicated by gray scale.
