# Supplementary material for: Tomato UDP-Glucose Sterol Glycosyltransferases: A Family of Developmental and Stress Regulated Genes that Encode Cytosolic and Membrane-Associated Forms of the Enzyme
Source: Front Plant Sci. 2017 Jun 9;8:984. doi: 10.3389/fpls.2017.00984 (PMC5465953; doi:10.3389/fpls.2017.00984)
Supplement: Supplementary file 5 [file Image_2.PDF]

Figure S2

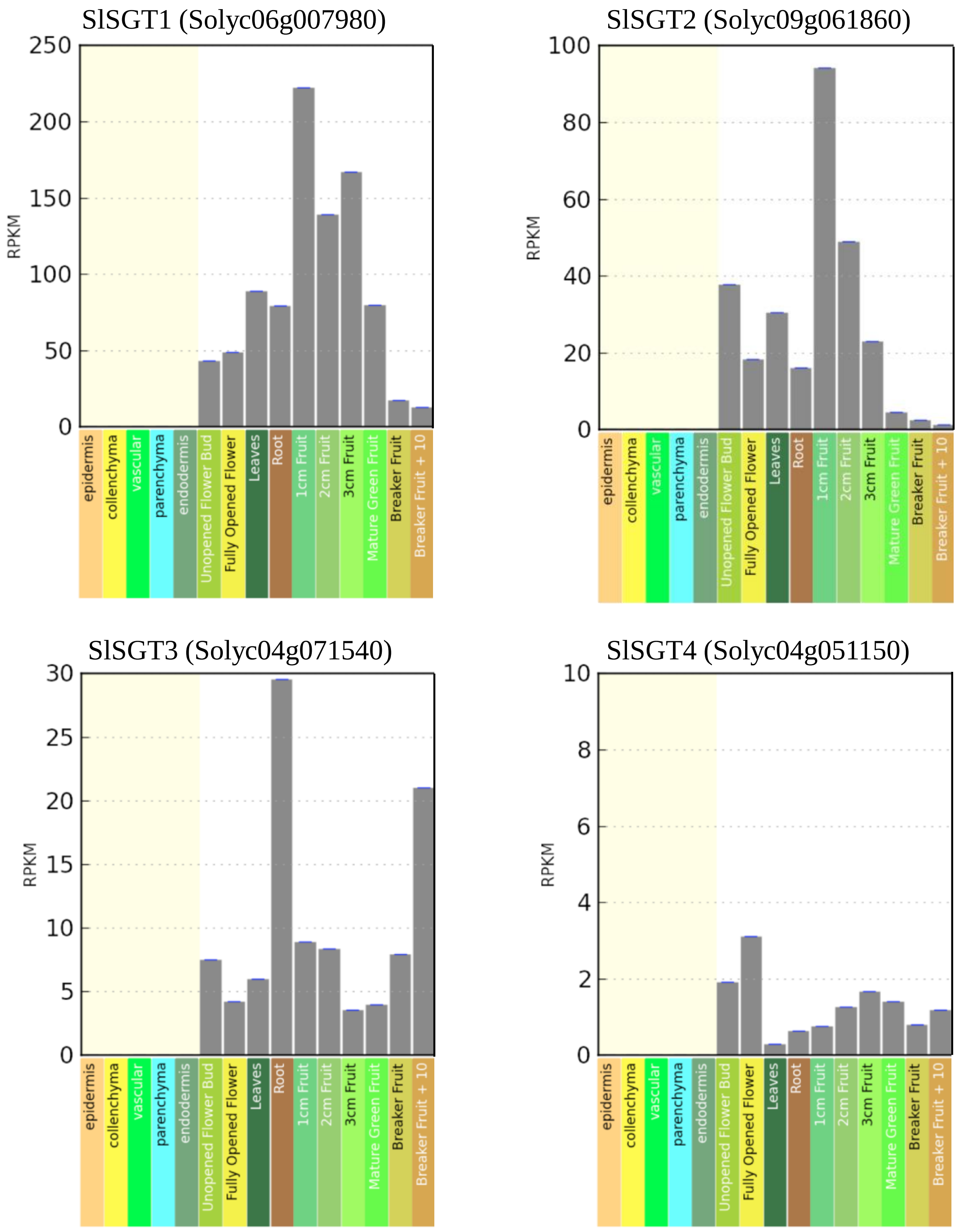

Figure S2. Screenshots from the tomato eFP browser (set at the Chart mode) showing the developmental expression of tomato *SGT* genes performed using RNA-seq data for *S. lycopersicum* cv. Heinz (http://bar.utoronto.ca/efp_tomato/cgi-bin/efpWeb.cgi).
