# Supplementary material for: Tomato UDP-Glucose Sterol Glycosyltransferases: A Family of Developmental and Stress Regulated Genes that Encode Cytosolic and Membrane-Associated Forms of the Enzyme
Source: Front Plant Sci. 2017 Jun 9;8:984. doi: 10.3389/fpls.2017.00984 (PMC5465953; doi:10.3389/fpls.2017.00984)
Supplement: Supplementary file 6 [file Image_3.PDF]

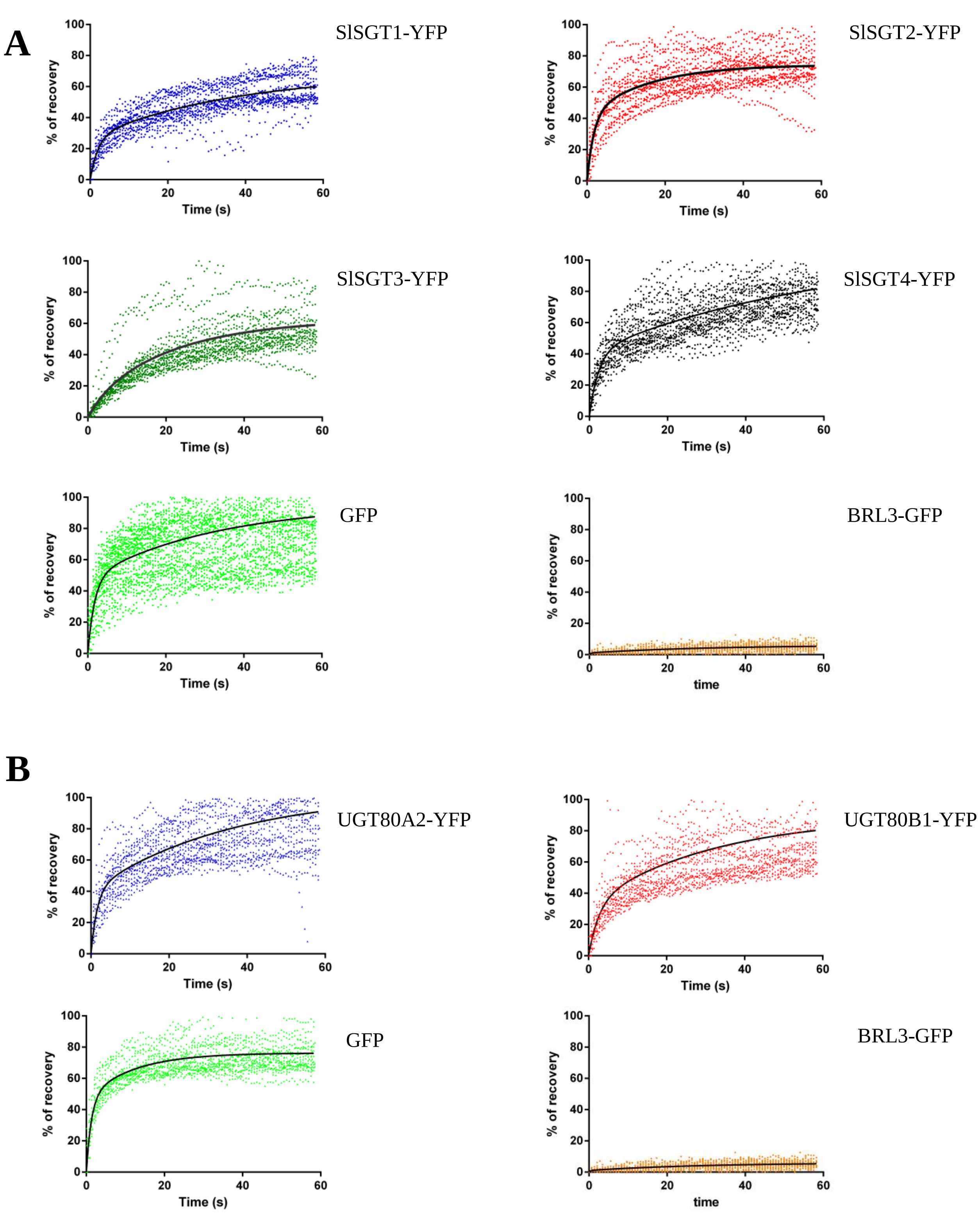

Figure S3. Complet data set of FRAP experiments. Plot showing each replicate (dots or triangles in color) used to create the best fit curve, black lines represent the best fit for each the dataset calculated using the two-phase exponential association equation. **A**. SlSGTs data set. **B.** UGT80A2 and UGT80B1
